# A scalable model for simulating multi-round antibody evolution and benchmarking of clonal tree reconstruction methods

**DOI:** 10.3389/fimmu.2022.1014439

**Published:** 2022-12-06

**Authors:** Chao Zhang, Andrey V. Bzikadze, Yana Safonova, Siavash Mirarab

**Affiliations:** ^1^ Bioinformatics and Systems Biology, University of California, San Diego, San Diego, CA, United States; ^2^ Computer Science and Engineering Department, University of California, San Diego, San Diego, CA, United States; ^3^ Electrical and Computer Engineering Department, University of California, San Diego, San Diego, CA, United States

**Keywords:** antibody evolution, clonal trees, joint tree and sequence evolution, birth-death models, method benchmarking

## Abstract

Affinity maturation (AM) of B cells through somatic hypermutations (SHMs) enables the immune system to evolve to recognize diverse pathogens. The accumulation of SHMs leads to the formation of clonal lineages of antibody-secreting b cells that have evolved from a common naïve B cell. Advances in high-throughput sequencing have enabled deep scans of B cell receptor repertoires, paving the way for reconstructing clonal trees. However, it is not clear if clonal trees, which capture microevolutionary time scales, can be reconstructed using traditional phylogenetic reconstruction methods with adequate accuracy. In fact, several clonal tree reconstruction methods have been developed to fix supposed shortcomings of phylogenetic methods. Nevertheless, no consensus has been reached regarding the relative accuracy of these methods, partially because evaluation is challenging. Benchmarking the performance of existing methods and developing better methods would both benefit from realistic models of clonal lineage evolution specifically designed for emulating B cell evolution. In this paper, we propose a model for modeling B cell clonal lineage evolution and use this model to benchmark several existing clonal tree reconstruction methods. Our model, designed to be extensible, has several features: by evolving the clonal tree and sequences simultaneously, it allows modeling selective pressure due to changes in affinity binding; it enables scalable simulations of large numbers of cells; it enables several rounds of infection by an evolving pathogen; and, it models building of memory. In addition, we also suggest a set of metrics for comparing clonal trees and measuring their properties. Our results show that while maximum likelihood phylogenetic reconstruction methods can fail to capture key features of clonal tree expansion if applied naively, a simple post-processing of their results, where short branches are contracted, leads to inferences that are better than alternative methods.

## 1 Introduction

Immune response to new pathogens relies heavily on the Affinity maturation (AM) process. AM follows the binding of immunoglobulin (IG) molecules to antigens and improves the affinity (i.e., binding ability) of B cells to the antigen (1, 2). The AM process involves many aspects, including the activation of naive B cells that have not been exposed to an antigen, clonal expansion of cells that increases the pool of antibodies, somatic hypermutations (SHMs) (3) that alter the structure of antibodies and their ability to bind, and a regulatory mechanism that plays the role of natural selection. The AM process creates memory and plasma B cells; memory B cells can be reactivated and can undergo the AM process again (4), while plasma B cells can secrete massive levels of neutralizing antibodies. Over time, the AM process leads to the formation of clonal lineages within a given antibody repertoire, where each clonal lineage is formed by descendants of a single naive B cell. The evolutionary history of each of these clonal lineages can be represented by a clonal tree, where each vertex corresponds to a B cell, and a directed edge connects each B cell to all its immediate descendants.

New sequencing technologies have enabled high-throughput scanning of antibody repertoires (AIRR-seq) and have opened up new avenues for studying adaptive immune systems (5–9). AIRR-seq technologies enabled AM analysis of antibody repertoires responding to antigens of various diseases, such as flu (10, 11), HIV (12, 13), hepatitis (14, 15), multiple sclerosis (16, 17), rheumatoid arthritis (18). Such analyses allow biologists to identify broadly neutralizing antibodies and reveal antigen-specific and general mutation patterns (11, 19).

**Figure 1:**
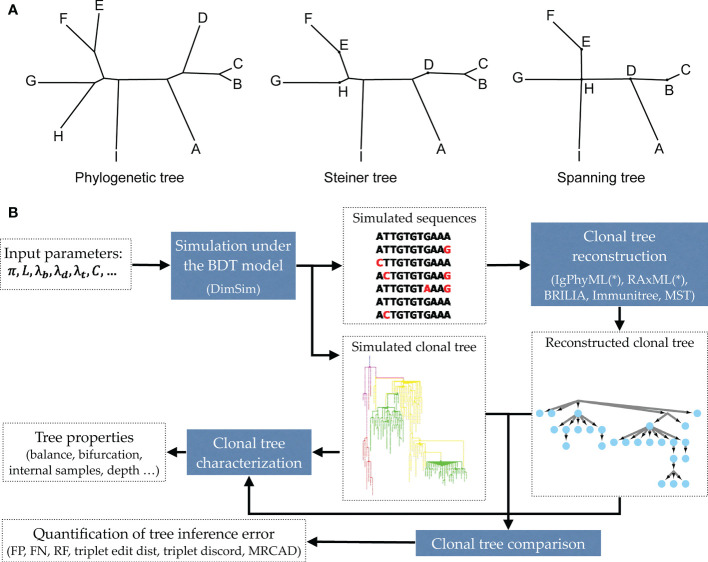
**(A)** Examples of a phylogenetic tree, a Steiner tree, and a spanning tree. Letters indicate sequenced data. Phylogenetic trees put all data points at leaves, and none at internal nodes, spanning trees put data at every node (whether internal or leaf), and Steiner trees are in between (some but not all internal nodes correspond to data). **(B)** The evaluation framework. The BDT model, parameterized by several values (**Table 1**) is first sampled using the fast algorithm implemented in DIMSIM to create the simulated (i.e., “true”) sequence data and clonal trees. These trees are then reconstructed from the simulated sequence data using various methods. The reconstructed clonal tree is compared to the simulated tree using several metrics adopted here to account for internal node sampling and multifurcation. Properties of true and inferred trees are measured using metrics such as balance and resolution.

Due to the short time frame of clonal expansion, inferred clonal trees have unique properties (20). Some sequenced nodes may belong to the internal nodes of the tree instead of the tips. Also, inferred clonal trees are often not even close to bifurcating. Thus, unlike traditional phylogenetics, perhaps Steiner trees (which can put observations at some of the internal nodes) or spanning trees (that put an observation at all internal nodes) should be preferred for reconstructing antibody sequences (**Figure 1A**). Various reconstruction methods have been developed attempting to recover clonal trees from antibody sequences [e.g., (13, 21–26)]. Some of these methods use simple clustering methods [e.g., (21)], while others formulate the problem as a Steiner tree problem (13, 22, 24, 26) or maximum-likelihood (ML) phylogenetic tree reconstruction under models of sequence evolution (23, 25).

In order to evaluate methods proposed for reconstructing clonal trees, we need models for antibody sequence evolution and clonal tree expansion that can be used for simulation. This modeling step is challenging for several reasons. (i) Selection, which is an integral part of AM, needs to be modeled directly; otherwise, the shape of the resulting trees will not be realistic. Traditional phylogenetics simulations first simulate a tree of sampled taxa and then evolve sequences down the tree. This two-step approach simplifies simulations but misses the dependency between the clonal tree shape and the antibody sequences. A better approach is to co-evolve the tree and sequences. The challenge in co-evolving is to design a principled model for how sequences impact evolution and to develop a scalable simulation algorithm that can generate large numbers of cells. (ii) Literature suggests that there are hotspots and cold spots of SHMs [e.g., (27, 28)]. However, traditional models of sequence evolution assume each site evolves independently and will miss the context dependence. (iii) Different antibody cell types (e.g., activated and memory cells) have very different mutational and selection behaviors, and these distinctions need to be modeled.

There have been several attempts at designing statistical models of AM clonal expansion [e.g., (20, 29–32)]. As the AM process is complex, these models have taken different routes. For example, determining affinities of sequences to hypothetical antigens is difficult, as affinity binding is a complicated chemical process, and each method models affinity differently. Nevertheless, all these methods have limitations, which we will return to in our discussion section. Two factors worth pointing out are that they do not scale to very large numbers of cells, and they allow for simulating one round of infection (as opposed to an evolving pathogen and recurring infections); some also avoid differentiating different types of B cells.

In this paper, we propose a scalable and flexible simulation framework that can be instantiated in many ways. We introduce a general birth, death, and transformation (BDT) model and describe how BDT can be instantiated to create a model of AM that simultaneously co-evolves the clonal tree and antibody sequences. We then introduce a scalable sampling algorithm for our model that enables generating large trees. With the simulator (called DIMSIM) at hand, we note that comparing clonal trees and characterizing their properties require care. We refine existing metrics and define new ones for characterizing properties (e.g., balance) of clonal trees and for comparing them. Finally, we perform extensive simulation studies (**Figure 1B**) under various parameters using DimSim. We study how the parameters of the AM model impact properties of clonal trees and benchmark the performance of several reconstruction methods.

## 2 Methods

### 2.1 Statistical models

We first define a general Birth/Death/Transformation (BDT) model and then present an efficient algorithm for sampling trees from that model. We then instantiate the general model for simulating AM processes and move on to describe specific choices we made in our simulations.

#### 2.1.1 BDT model

Forward-time birth-death models are used extensively in macro-evolutionary modeling (33), whereas microevolution simulations often use coalescent models that are easier to sample. We propose a general forward-time model that can allow realistic microevolutionary simulations by ensuring that birth and death rates are not constant and instead change with the properties of evolving units (e.g., cells).

In the BDT model, a set of entities continuously undergo birth (B), death (D), and transformation (T) events. Each entity i has a list of properties ${\text{x}}_{i}\in {\mathbb{R}}_{+}^{N}$ . At each point in time, the system contains a set *S* of *n* active entities, and each active entity *i ∈ S* undergoes birth, death, and transformation events according to independent Poisson point processes. In the birth event, an entity *i* is removed from *S* and new entities *j* and *k*, with properties **x** *_j_* and **x** *_k_*, are added to *S*; properties **x** *_j_* and **x** *_k_* are drawn from a distribution determined by **x** *_i_* and model parameters. In the event of the death of an entity *i*, it is removed from *S*. In the transformation event, an entity *i* is removed from *S* and a new entity *j* with properties **x** *_j_*, drawn from a distribution determined by **x** *_i_*, is added to *S*. Starting from a single node and continuously applied, this process defines a rooted tree where nodes are all entities that ever existed (including those that died); birth events create bifurcations, transformation events create nodes with one child, and death events create leaves. The tree can be subsampled subsequently.

For each entity *i ∈ S*, the birth, rate, and transformation rates are thoroughly determined by its properties **x** *_i_* and the sum of properties over all entities **S** = Σ_*j*∈*S*_ **x**_*j.*_ We let Λ_*B*_(**x**_*i*_, **S**), Λ_*D*_(**x**_*i*_, **S**), and Λ_*T*_(**x**_*i*_, **S**) denote the birth, death, and transformation rates, respectively. In the time interval between two events for any two entities in the system, we assume a memoryless process. Thus, these rates remain constant between any two events but can change when an event happens. The ratio between the birth rate and the death rate, both of which are functions of the entity properties, can be thought of as the factor controlling the selective pressure, which can be time-variant.

Because of the memoryless property, the time until the next BDT event always follows the exponential distribution with rates Λ_*B*_(**x**_*i*_, **S**), Λ_*D*_(**x**_*i*_, **S**), and Λ_*T*_(**x**_*i*_, **S**) for each event type. The time until any event for any entity follows an exponential distribution with *λ* = Σ_*i*∈*S*_ (Λ_*B*_(**x**_*i*_, **S**) + Λ_*D*_(**x**_*i*_, **S**) + Λ_*T*_(**x**_*i*_, **S**)). The probability of the next event being a specific event *E ∈ {B, D, T}* for a particular entity *i* is Λ*_E_* (**x** *_i_*, **S**)*/λ*. Specifying the rate functions and the distribution of properties at the initial state fully specifies the model.

The BDT model can be efficiently sampled if rate functions have certain (very general) properties. We leave all the mathematical details for **Appendix 2.1**. In short, the memoryless property of the model makes it possible to perform efficient sampling despite the fact that rates change with the tree. The main innovations of the sampling algorithm are 1) rewriting rate functions as polynomial functions of other parameters, which enable finding the time to the next event in constant time, and 2) using an interval tree data structure to store partial sums needed for normalization. With our proposed **Algorithm S1** (in **Appendix 4**), a tree on *k* nodes drawn from the distribution defined by the BDT process can be sampled in *O*(*k* log(*k*)) time. Thus, the BDT model can be efficiently sampled to create trees with millions of nodes.

#### 2.1.2 Antibody affinity maturation model

We now define a specific instance of the BDT model designed for AM. Simulations according to this AM model are implemented in a C++ tool called Dynamic IMmuno-SIMulator (DIMSIM). The model has many parameters reflecting immune system properties (**Table 1**), which we define throughout this section (**Appendix 1** defines our particular usage of terms commonly used in immunology). The use of birth death models for AM is not new [e.g., (32)] but particular choices of our model are different from prior work.

**Table 1:** Parameters of the AM model.

Param.	Default	Parameter description
$\lambda_{d}^{'}$	1/402	Rate (inverse life time) of cell death for memory cells (days^*−*1^)
*λ_b_*	6	Rate of cell division for activated B cells (days^*−*1^)
*λ_d_*	10^4^	Rate of cell death during dormant stage (day^*−*1^).
*λ_t_*	0.01	Rate of activation of a typical responsive memory cell
*ρ_p_*	1*/*100	Portion of activated B cells that turn into plasma cells per cell division
*ρ_m_*	1*/*4	Portion of activated B cells that turn into memory B cells per cell division
*µ*	5 *×* 10^−4^	Rate of SHMs per base pair per generation
**K** ^5^	**Appendix 2**	Empirical 5-mer mutation frequencies per generation
*L*	125	Length of the amino acid antibody-coding sequence (assuming the length is fixed)
**CDR**	31–35,50–65, 98–114	Positions of the three CDR regions (amino acid coordinates)
*δ*(*i, j*)	**Table S2**	BLOSUM matrix defined on a pair of amino-acids *i* and *j*
Δ_0_	-120	BLOSUM score of a typical memory B cell antibody-coding sequence to target
$\Delta_{0}^{'}$	-75	BLOSUM score of activated B cell antibody-coding sequences that leads to cure
*w_f_*	1*/*3	BLOSUM score multiplier of non-CDR positions (i.e., FRs)
*κ*	2	BLOSUM score ratio of antibody-coding sequences to antigen sequences
*A*	0.1	Selective pressure: factor connecting sequence similarity and log binding affinity
*ρ_a_*	1*/*2	Factor connecting log affinity and B cell activation (sensitivity to affinity level *A*)
*C*	10^5^	Carrying capacity limited by total resources (see text for meaning)
*M*	$Ce^{A\Delta_{0}^{'}}$	The threshold of the sum of affinity for a stage change
*r*		Rounds of viral infections
$\hat{\Psi }$	**Appendix 2**	Nucleotide sequence of the initial B cell
***ζ***_1_… ***ζ*** *_r_*	**Appendix 2**	Target amino acid sequences for viral infections in each round
***η***_1_… ***η*** *_r_*	**Appendix 2**	Flu sequences assumed as antigens in the simulation
*t*_1_… *t_r_*	**Appendix 2**	Starting time of each infected stage (day)

##### 2.1.2.1 Rounds and stages

We model the evolution of antibody-coding sequences in response to *r* rounds of infections by an evolving antigen (e.g., SARS-Cov2 or flu). Each round consists of two stages, an infected stage, where a set of new antigens initiate a response that activates the B cells being modeled, and a dormant stage, where the B cells being modeled are not actively involved in an immune response. Both stages used the same BDT model but are parameterized differently. The switch between the two stages happens through user-defined rules (e.g., rules that reflect infection progression as described below). During the infected stage of round *i*, we target amino-acid sequence ζ_i_ = (ζ_*i*_^(1)^, …, ζ_*i*_^(*L*)^) of length L without any stop codon, defined as the best possible antibody-coding sequence that can bind to the present antigen. The target can change across rounds, and we describe two specific ways of choosing targets in Section 2.1.3.

##### 2.1.2.2 Cell properties

**Figure 2:**
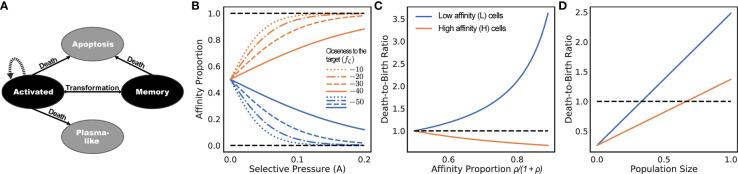
**(A)** States of cells and transitions during infected stage. Only states colored black are modeled. Transitions to states colored gray are treated as death events. **(B-D)** Consider a population of activated B cells where all cells have one of two sequences: L (low) or H (high). Let *ρ* be the ratio of affinity of H-type cells to L-type cells, and let the affinity proportion be the total affinity of a cell type over the total affinity (i.e., *ρ/*1 + *ρ* for H and 1*/*1 + *ρ* for L). **(B)** The affinity proportion as a function of the selective pressure *A* when the sequence closeness to the target *f_ζ_* (.) is kept fixed for L and varies for (H). **(C)** the ratio of death rate to birth rate as a function of affinity proportion of H cells, fixing the population size to the carrying capacity. **(D)** Ratio of death rate to birth rate as a function of the population size normalized by the carrying capacity, fixing *ρ* = 2. All other parameters set to defaults (**Table 1**). The selective pressure *A* and the level of binding control the portion of affinity taken up by better sequences **(B)**, which controls the growth of the cell type **(C)**, which is also a function of the total population size **(D)**.

Since only memory B cells can be repeatedly activated by an encounter with antigens, we only simulate memory B cells. Plasma B cells do not undergo SHMs and represent terminal states of the clonal lineage development and thus can be sampled from the leaves of the simulated tree. We will refer to a B cell that has just encountered an antigen and moved to a germinal center (GC) as an activated B cell (or “activated cell” for short) (**Figure 2A**).

In the AM model, each entity *i* represents a B cell with the property vector **x** *_i_* = (*g_i_, s_i_, t_i_, g_i_/a_i_, g_i_a_i_*) with five values, among which the last three are derived from two other quantities: *gi* and *a_i_* (only saved in the derived form). We keep derived properties as part of **x** *_i_* because they allow us to define Λ*_E_* (**x** *_i_*, **S**) as polynomials of saved properties; this, in turn, enables the use of our fast sampling algorithm. To follow the BDT model, we assume the properties of each cell are fixed in between B/D/T events, ignoring possible short-term temporal changes (34). We next define *g_i_, s_i_, t_i_*, and *a_i_*.

- The binary property *g_i_* indicates whether a cell *i* is an activated B cell (1) or is a memory B cell outside lymph nodes, which we call a “memory cell” for simplicity (0).
- The *s_i_* property stores the DNA sequence of B cell *i* coding for the variable region of the heavy chain with a fixed length 3*L*. We focus on simulating the heavy chain sequences only because most existing AIRR-seq studies focus on sequencing heavy chains only [e.g., (11, 16, 35, 36)]. For the sake of simplicity, we assume the fate of the cell depends only on the variable region of the heavy chain. Each cell has a fixed sequence, and mutations occur at the time of a cell birth, which happens only for activated cells in the infected stage. After a birth event for cell *i*, sequences *s_j_* and *s_k_* of child cells *j* and *k* are chosen independently and identically at random [ignoring the G1 origin of mutations (37)]. While any sequence evolution model could be incorporated in the DIMSIM framework, in Section 2.1.3, we describe a 5-mer-based model used in our analyses.
- Property *t_i_* denotes the rate of transformation, which means the activation of a memory cell (*g_i_*: 1 *→* 0) in response to an antigen, or the maturation of an activated cell into a memory cell (*g_i_*: 0 *→* 1). Transformations, which only happen during the infected stage, flip *g* but keep the sequence *s* intact.
- Property *a_i_* denotes the strength of affinity binding of the Ig receptor of the cell *i* to the antigen.

We let *σ* denote the total affinity of activated cells and note *σ* denote the total affinity of activated cells and note *σ* = Σ_*i*∈*S*_ *g_i_a_i_* is the last element of the vector **S**. Then, *a_i_/σ* is the fraction of total affinity assigned to a cell. Both *t_i_* and *a_i_* are derived and are set based on the sequence of *i* and the target.

##### 2.1.2.3 Sequence affinity and birth, death, and transformation rates

Affinity *a_i_* is only defined and used during the infected stage where the target is available and is function of the cell sequence *s_i_* and the target sequence *ζ.* The closer the sequence to the target, the higher its affinity should be, a fact that other simulators have also incorporated (30, 32). The exact relationships between the sequences and affinity are not known. For the purpose of benchmarking methods, we propose a simple formula. Let *f_ζ_* (*s_i_*) be a measure of the closeness of the sequence to the target in the affinity space, we set


(1) $$a_{i}≐e^{Af_{\varsigma }(s_{i})}$$

where *A* is a constant factor used to calibrate the selective pressure. As sequences get closer to the target, the affinity grows gradually with a speed controlled by *A* (**Figure 2B**). We will describe our particular choice of function *f_ζ_* (*s_i_*) using BLOSUM similarity in Section 2.1.3.

The event rates are functions of cell properties and the stage (**Table 2**). During the dormant stage, there are no births or transformations; cells only die with a very high uniform rate *λ_d_* for activated cells and a low uniform rate $\lambda_{d}^{'}$ for memory cells.

**Table 2:** Birth, death, and transformation rates.

Rate functions	Infected stage	Dormant stage
Λ*_B_* (**x** *_i_*, **S**)	*g_i_λ_b_* + (1 *− g_i_*) *×* 0	0
Λ*_D_* (**x** *_i_*, **S**)	$g_{i}(\frac{\lambda_{b}(1-\rho_{p}-\rho_{m})}{C}\frac{\sigma }{a_{i}}+\rho_{p}\lambda_{b})+(1-g_{i})\lambda_{d}^{'}$	$g_{i}\lambda_{d}+(1-g_{i})\lambda_{d}^{'}$
Λ*_T_* (**x** *_i_*, **S**)	$t_{i}=g_{i}\rho_{m}\lambda_{b}+e^{-\rho aA\Delta_{0}}a_{i}^{\rho a}(1-g_{i})$	0

See **Table S1** for polynomial forms.

During the infected stage, we adjust the death rates of cells based on their affinities but keep the birth rates constant; this interplay is used to simulate the selective pressure. Note that we do not claim that a fixed birth rate and changing death rate is biologically realistic [e.g., see (38)]. However, in terms of the dynamics of our model, what matters is the ratio of the birth and death rates, which enable us to make this simplifying choice. In our model, an activated cell can undergo cell division at a uniform rate *λ_b_*, differentiate into a memory cell at a uniform rate *t_i_* = *ρ_m_λ_b_* or a plasma-like cell at a uniform rate *ρ_p_λ_b_*, and undergo apoptosis (i.e., die). We do not model plasma-like cells; instead, both differentiation into plasma-like cells and apoptosis are treated as death events (**Figure 2A**). The rate of apoptosis of an activated cell *i* is modeled as inversely proportional to the amount of resources (antigens and FDCs) to which the cell *i* has access when competing against other activated cells. Thus, the proportion of resources available to the cell *i* is modeled by the affinity proportion *a_i_/σ* (i.e., the affinity of the cell to the antigen normalized by the current sum of the affinity of all activated cells). This affinity proportion is impacted by the parameter *A*. The lower the *A*, the more uniform these proportions become, modeling low selective pressure; conversely, as *A* increases, *a_i_/σ* values further diverge between low affinity and high-affinity cells (**Figure 2B**). Thus, *A* can be used to control the strength of the selective pressure.

The memory cells undergo apoptosis at a uniform rate $\lambda_{d}^{'}$ . They can also be activated by helper T cells to enter the germinal center with the transformation rate


(2) $$t_{i}≐\lambda_{t}e^{\rho_{a}A(f_{\varsigma }(s_{i})-\Delta_{0})}=\lambda_{t}e^{-\rho_{a}A\Delta_{0}}a_{i}^{\rho_{a}}$$

Note that the activation rate of memory cells increases monotonically with their affinity to the target, according to $a_{i}^{\rho_{a}}$ where *ρ_a_*, set by default to 1*/*2, is the sensitivity of B cell activation to affinity. This dependency on affinity models the increased propensity of the memory cells to activate when presented by helper T cells with familiar antigens. The default choice *ρ_a_* = 1*/*2 is motivated by the fact that although memory cells with higher binding strengths to the antigen are more likely to be activated, the interaction between a helper T cell and a memory B cell is a one-time event and thus less sensitive to binding strength.

As an example, consider a system with two cell types: L and H, each type with its own unique sequence (**Figures 2B-D**). Assume all cells are activated cells, the number of L and H are the same at one point in time, and H cells have a higher affinity than L cells by a factor of *ρ*. For ease of exposition, here, we include the mutation rate as part of the death rate because mutation events also decrease cell count. Let’s assume the total number of cells equals the carrying capacity *C*. If L and H have the same affinity (i.e., *ρ* = 1), then the birth and death rates are identical for all cells. As the affinity of H cells is increased (i.e., *ρ >* 1), the death rate of L cells increases linearly, whereas the death rate of H cells decreases (**Figure 2B**). Thus, H cells will have higher birth rates than death, will be selected for, and will expand. If we fix *ρ* = 2 and increase the population size, the death rates of both L and H cells increase, but at different rates (**Figure 2D**). When the population size is small compared to *C*, both types of cells have more birth than death. After a threshold (*C/*3 in this example), the death rate of L type surpasses its birth rate (thus, its population starts to shrink) while the population of H cells continues to grow. However, as the population size increases (2*C/*3 here), both sets of cells start to shrink (i.e., higher death rates than birth), because the population size is by definition bounded by *C.*

#### 2.1.3 Specific modeling choices

Several steps of our simulations are flexible and can be changed by the user. We next describe the set of choices we have implemented and used in the experiments below. We have performed two sets of experiments with different settings; where the two simulations differ, we explain both choices.

*Switching between stages*. The system enters dormant stage when antigens are neutralized by the antibodies. We have implemented two options for defining neutralization. *i*) Switch to the dormant stage after a certain duration from the beginning of the infected stage. *ii*) Switch to the dormant stage when the total affinity of antibodies produced by plasma-like cells reaches a certain threshold; here, we switch when the sum of affinities of activated cells (*σ*) reaches a predefined constant *M.*

*Sequence evolution*. In our experiments, we use an empirical 5-mer-based model inspired by the Yaari et al. (39) model. Let *s*(*p*) be the nucleotide on the *p*-th position of the nucleotide sequence of the cell *i*. Each $s_{j}^{\left(p\right)}$ or $s_{k}^{\left(p\right)}$ is independently set to *s ∈* {*A, C, G, T*} with probability: $Pr(s_{j}^{\left(p\right)}=s)=Pr(s_{k}^{\left(p\right)}=s)=f(s,s_{i}^{\left(p-2\right)},s_{i}^{\left(p-1\right)},s_{i}^{\left(p\right)},s_{i}^{\left(p+1\right)},s_{i}^{\left(p+2\right)})$ where *f*: {*A, C, G, T*}^6^→ [0, 1] denotes an empirically determined 5-mer frequency model based on the Yaari et al. (39) model and recomputed based on newer datasets including non-synonymous mutations (**Appendix 2**). Note that the rate of the mutation of each position changes as the sequence around it changes (e.g., from a low rate 5-mer to a high rate 5-mer).

*Sequence affinity function*. While various methods can be imagined for measuring the closeness of the sequence to the target, we used a simple approach: measuring sequence similarity according to the BLOSUM matrix and appropriate scaling of numbers. We assume each amino-acid position contributes to the binding strength to the target and the stability of the structure of the Ig-receptor independently. We model affinity proportionally to the product of the effect of each amino-acid position. This simple model ignores the 3D structure of proteins for the most part but should be sufficient for creating benchmarking datasets as none of the reconstruction methods consider 3D structure either. However, because complementarity-determining regions (CDRs), which include the binding sites, tend to accumulate more SHMs compared to framework regions (FRs) (19, 40), we do differentiate those. When *s_i_* includes a stop codon, we simply set *a_i_* = 0. Otherwise, we define the BLOSUM score of an amino acid sequence *ξ* = (*ξ* ^(1)^ *,…., ξ* ^(*L*)^) with respect to target *ζ* as


(3) $$\Delta_{\varsigma }(\xi )=\sum_{p\in \text{CDR}}\left(\delta (\xi^{\left(p\right)},\varsigma^{\left(p\right)}\right)-\delta (\varsigma^{\left(p\right)},\varsigma^{\left(p\right)}))+w_{f}\sum_{p\in \{1...L\}\backslash CDR}\left(\delta (\xi^{\left(p\right)},\varsigma^{\left(p\right)}\right)-\delta (\varsigma^{\left(p\right)},\varsigma^{\left(p\right)}))$$

where *δ*(*.,.*) gives the BLOSUM score between two amino acids (**Table S2**), and *w_f_* is a constant used to calibrate the importance of CDRs versus FRs in the affinity and transformation processes. We then simply set *f_ζ_* (*s_i_*) = Δ*_ζ_* (*ξ*(*s_i_*)) where *ξ*(.) translates from DNA to AA.

*Choosing targets*. One target sequence per round needs to be selected. The extent of the change in targets across rounds impacts the patterns of the immune response and hence the shape of the resulting clonal trees. To define targets across rounds in our experiments, we explore two options. *i*) We simply use the antibodies known to neutralize a specific disease as the target. Existing databases such as CoV-AbDab (41) provide excellent sources of such candidates. *ii*) Evolutionary approach: We seek a set of sequences with an evolutionary trajectory that reflects the evolutionary history of a set of real antigens (e.g., influenza virus). Let the known amino-acid sequences of an antigen (e.g., flu) sampled through time be denoted by ***η***_1_ *,…*, ***η*** *_r_*, and let each sequence have the fixed length *L_η_.* To choose the targets, we first select an arbitrary naive B cell, here chosen from datasets of Ellebedy et al. (35), and set $\hat{\Psi }$ to the nucleotide sequence of the variable region of its heavy chain.

Then, we simply set ***ζ***_1_ to the amino-acid translation of $\hat{\Psi }$ . In other words, in the first round, we use the naive cell as the target, and therefore, the first couple of rounds of the simulation should be treated as dummy rounds and should be discarded. Let *κ* be a positive constant that controls the rate of change in the target relative to the rate of change in the antigen sequences. To define the remaining targets, we seek to find the set of *r −* 1 sequences that minimize:


(4) $$\sum_{i,j\in [r]}|\kappa \sum_{p\in \text{CDR}}\delta (\varsigma_{i}^{\left(p\right)},\varsigma_{i}^{\left(p\right)})-\delta (\varsigma_{i}^{\left(p\right)},\varsigma_{j}^{\left(p\right)})-\sum_{q=1}^{L_{\eta }}(\delta (\eta_{i}^{\left(q\right)},\eta_{i}^{\left(q\right)})-\delta (\eta_{i}^{\left(q\right)},\eta_{j}^{\left(q\right)}))|.$$

Thus, a set of target sequences across *r* rounds are preferred if their pairwise distance matrix maximally matches the pairwise distance matrix of all antigen sequences over the same rounds (with a scaling). To account for conserved regions, we arbitrarily chose to keep all the non-CDR regions invariable in all target sequences (this choice can be easily changed). Thus, we seek to make the distance between two target sequences from two rounds similar to the distances of antigen sequences, scaled by a factor of *κ*. We approach this NP-hard problem using a greedy search heuristic (**Algorithm S3**). The heuristic starts with arbitrary *ζ*_2_ *,…, ζ_r_* and replaces one symbol of one sequence at a time to reduce the objective function; it repeats until reaching a local minimum where no such replacement is possible.

### 2.2 Benchmarking setup

We use two sets of experiments, one focused on SARS-CoV2 and one focused on influenza. The two sets of simulations use different settings to demonstrate the flexibility of the tool. In both experiments, unless otherwise specified, we used the default settings for the various parameters of **Table 1**.

#### 2.2.1 SARS-CoV2 simulations

We performed 3–5 rounds of infections by SARS-CoV2. For SARS-CoV2, the potential antibodies neutralizing the virus are available, providing us with a natural way to choose the targets. We selected all heavy chain sequences of human antibodies with IGHV1-58 and IGHJ3 from the Coronavirus Antibody Database (41) that neutralize some variants of SARS-CoV2 and have 16 amino acids in their CDR3. We kept only one sequence per upload date by choosing the antibody that neutralizes the most variants of SARS-CoV2 and obtained 14 sequences (**Table S3**). We then simulated 3–5 (randomly selected) infections with targets randomly selected from the sequences in **Table S3** and the infection start date set to be the upload date. We ensured that gaps between the start dates of any two infections were more than 50 days. Each round of infections is set to last 50 days. We repeat this process for 50 replicate individuals.

For each individual, at the end of simulations, we sample *ς* = 50, 100, 200, 500 antibody-coding nucleotide sequences from cells in the system (i.e., from the last round of infection) and built their clonal tree. We also examine scaling of a subset of methods by testing ς ∈ {50, 100, 200, 500}.

#### 2.2.2 Flu simulations

We performed several simulations of a series of *r* = 56 rounds of flu, using sequences of hemagglutinin (HA) protein. HA found on the surface of the influenza viruses is the primary target of neutralizing antibodies. High mutation rates of influenza genome change the sequence of HA and allow the virus to escape from the immune pressure, thus making flu a recurring seasonal infection. The NCBI Influenza Virus Resource (42) contains 961 HA sequences from influenza B virus collected around the world. Each HA sequence is labeled with a year and a location. For simulation purposes, we extracted 59 HA sequences corresponding to flu infections in Hong Kong and selected 56 out of 59 HA sequences with the same length (584 aa). The selected HA sequences were detected in Hong Kong from 1999 to 2010. Notice that HA sequences could be replaced with other widely available antigen sequences (e.g., Coronavirus).

We used the evolutionary approach described earlier to choose the target amino-acid sequences. Each round corresponds to one season, starts at the infected stage with a given target sequence ***ζ*** *_l_*, and ends when *σ* = *M.* At that point, we assume the infection is overcome, and the system switches to dormant, where we stay until the next round starts (times of flu outbreaks are known in our dataset). When the *r* = 56 rounds of infections end, we sample *ς* = 200 antibody-coding nucleotide sequences Ψ_1_ *,…*, Ψ*_ς_* from cells in the system (i.e., from the round *r*) and built their clonal tree. While it is unrealistic for a person to get infected with flu these many times, this procedure allows us to test the impacts of a large number of infections.

Here, we set up four experiments, varying one or two parameters in each experiment (**Table 3**) and setting the remaining ones to default values (**Table 1**). The central experiment contains 19 conditions, changing the selective pressure (*A*) and the rate of hypermutation (*µ*). We vary *A* from 1*/*8*×* of default value (0.1) to 2*×* and vary *µ* s from 1.25 *×* 10^*−*4^ to 2 *×* 10^*−*3^ per base-pair per generation. In six combinations, the selective pressure is not high enough to overcome random mutations; in these cases, the affinity values do not increase, and as a result, the carrying capacity is never reached. Thus, we exclude these conditions. We also study three other parameters. We vary the weight multiplier of FRs (*w_f_)* from 1*/*5 to 2. We vary the carrying capacity (*C)*, which is the germinal center size or the amount of antigens FDCs hold in the context of B cell maturation, from 12500 to 400000. The value of this parameter can impact the speed of novel mutations arising and may change the properties of simulated trees. We also vary the mean lifetime of memory cells from 0.5 year to 16 years to study the impact of the extent of memory cell activation during recurrent infections.

**Table 3:** Experiment setup.

Experiment	Parameters	Parameter values	Parameter units
Selective pressure *vs*. rate of hypermutation	*A × µ*	(2, 2), (2, 1), (2, 1*/*2), (2, 1*/*4), (2, 1*/*8), (1, 2),	*A*: 10^*−*1^
		(1, 1), (1, 1*/*2), (1, 1*/*4), (1, 1*/*8), (1*/*2, 1), (1*/*2, 1*/*2),	*µ*: 10^*−*3^
		(1*/*2, 1*/*4), (1*/*2, 1*/*8), (1*/*4, 1), (1*/*4, 1*/*2),	
		(1*/*4, 1*/*4), (1*/*4, 1*/*8), (1*/*8, 1*/*4), (1*/*8, 1*/*8)	
Framework weight	*w_f_*	2, 1, 1*/*2, 1*/*3, 1*/*5	1
Germinal center size	*C*	4, 2, 1, 1*/*2, 1*/*4, 1*/*8	10^5^
Memory cell life	1/ $\lambda_{D}^{'}$	16, 8, 4, 2, 1, 1*/*2	year (365 days)

#### 2.2.3 Compared methods of clonal lineage reconstruction

We compare eight tools: minimum spanning tree, BRILIA (22), IgPhyML (23), RAxML (43), Immunitree (13), Dnapars (44), BEAST (45), and GCtree (26). We also include an alternative “contracted” version for four methods (IgPhyML*, RAxML*, BEAST*, Dnapars*). We note this is not an exhaustive list, as many other tools also exist [e.g., SAMM (32) and IgTree (46)] that we did not include. For all methods, we ran reconstructions using one CPU core and set a 24-hour wall time for each replicate.

MST(-like) methods: We implemented a simple minimum spanning tree method containing Ψ_1_ *,…*, Ψ*_ς_* as well as $\hat{\Psi }$ which is forced to be the root. We compute the nucleotide Hamming distance between all pairs of sequences and construct the minimum spanning tree (MST) using those distances. Besides the simple MST, we also test Immunitree (13), a tool that clusters antibody-coding sequences into lineages and builds clonal trees at the same time by optimizing a minimum spanning tree and Steiner tree-like problem. We took as input Ψ_1_ *,…*, Ψ*_ς_* and used Immunitree to build a set of clonal trees. We then added vertex $\hat{\Psi }$ as the root and let the roots of the clonal trees to be immediate children of $\hat{\Psi }$ .

Brilia clusters antibody-coding sequences into lineages and builds clonal trees at the same time. We took as input Ψ_1_ *,…*, Ψ*_ς_* and used Brilia v3.5.4 to build a set of clonal trees. We then added vertex $\hat{\Psi }$ as the root and added roots of the clonal trees as children of $\hat{\Psi }$ .

Phylogenetic methods: We tested ML phylogenetic reconstruction tool RAxML v8.2.12 under the GTR+GAMMA model and IgPhyML v1.1.5, an ML method tuned specifically for immune cells. We also tested maximum parsimony (MP) trees using Dnapars from the PHYLIP package v3.697 (44) and Bayesian MCMC using BEAST v1.10.4 (45). We ran BEAST under the GTR+GAMMA model and uncorrelated relaxed clock with log-normal distribution; we set the tree prior model to Bayesian Skyline, the length of MCMC chain to be 108, and the burn-in rate to 10%. For RAxML, Dnapars, and BEAST, we took as input Ψ_1_ *,…*, Ψ*_ς_* and $\hat{\Psi }$ to obtain an unrooted phylogenetic tree and rerooted at $\hat{\Psi }$ . For Dnapars, we reported the greedy consensus tree when Dnapars outputted multiple trees. We also report the majority consensus tree and refer to it as Dnapars*. For IgPhyML, we took as input Ψ_1_ *,…*, Ψ*_ς_* and provided $\hat{\Psi }$ as root to obtain a rooted phylogenetic tree. RAxML, IgPhyML, and BEAST produce fully binary trees, while Dnapars can produce non-binary trees.

Contracted phylogenetic methods: As previously suggested (26, 32), contracting short or low support branches is one way of addressing the limitations of methods that output fully binary trees. Since the length of each antibody-coding nucleotide sequence < 400, we can assume that both ends of any branch with length less than 10^*−*4^ would correspond to the same sequence (if it was sampled). Therefore, we contracted branches of length less than 10^*−*4^ for RAxML and IgPhyML, and call the resulting methods RAxML* and IgPhyML*. While the same logic does not hold for the Bayesian methods (because of the prior on branch lengths), for consistency, we applied the same procedure to BEAST to obtain BEAST*.

GCtree is an approach that integrates sequence abundance information to break ties in an MP analysis (26). It allows sequences to be placed on internal nodes. We took Ψ_1_ *,…*, Ψ*_ς_* as input to GCtree v4.04 and provided $\hat{\Psi }$ as root to obtain a rooted phylogenetic tree. As Ψ_1_ *,…*, Ψ*_ς_* often contain repetitive sequences, GCtree can utilize the sequence frequencies for lineage reconstruction.

#### 2.2.4 Evaluation metrics

The simulated and reconstructed histories of samples Ψ_1_ *,…*, Ψ*_ς_* are represented as trees, where samples are uniquely labeled on some nodes, and the remaining nodes are left unlabeled. Labeled nodes represent sequences in the samples, and unlabeled nodes denote the ancestral sequences not present in the samples. We evaluate results in two ways, described in detail in **Appendix 2.4**. We use a set of metrics for characterizing properties of simulated trees in terms of their topology, branch length, and distribution of labeled nodes. We also compare the simulated trees to those inferred using each method (**Table 4**).

**Table 4:** Clonal tree properties and metrics for comparing the reference tree *R* to estimated tree *E*.

Property/Metric	Definition
Internal sample (%)	The percentage of labeled nodes that are internal nodes.
Bifurcation index	Ratio of the number of internal nodes to one less than labeled nodes; equals to 1 for bifurcating trees and ≈ 0 for a star tree.
Sample depth	The average depth of labeled nodes.
Balance (cherry)	Half the sum over all leaves of the fraction of their siblings that are also leaves.
Single mutation branches (%)	The percentage of branches with length one.
Accumulated mutations (avg)	The average depth (path length to the root) of all labeled nodes.
Accumulated mutations (sum)	The summation of branch lengths of all branches.
Mutations per branch	The average branch length.
False Discovery Rate (FDR)	the percentage of clusters in *E* that are not in *R* False
Negative Rate (FNR)	the percentage of clusters in *R* that are not in *E* RF
cluster distance (RF)	the number of clusters in either but not both trees
FDR*, FNR*, and RF*∗*	*: similar to the previous metrics but with singletons excluded
Triplet discordance (TD)	the number of trees induced by triples of labeled nodes (leaf or internal) where the topology in the simulated tree and the reconstructed tree differ
Triplet edit distance (TED)	the sum of the RF cluster distance induced to each triplet of labeled nodes
MRCA Discordance (MD)	the summation of MRCA discordance*†* over all ordered pairs of labeled nodes.
Patristic Distance (PD)	the summation of the patristic discordance*‡* over all pairs of labeled nodes.

†MRCA discordance of two labeled nodes is the difference between the number of branches in the path between each of them and their MRCA.

‡Patristic discordance for a pair of labeled nodes is the difference between the number of branches in the path between the two nodes on the two trees *R* and *E*. See also **Table S6**.

While metrics for comparing phylogenies exist, these metrics need to be amended for clonal trees that can have sampled ancestral nodes (32, 47). Many of the existing metrics can be generalized to compare a simulated tree *R* and a reconstructed tree *E* (**Table 4**), both induced down to include all labeled nodes (i.e., removing unlabeled nodes if less than two of their children have any labeled descendants). Unlike traditional phylogenies, here, internal nodes can be labeled, and we define metrics based on rooted trees instead of unrooted trees. We refer to the set of labeled nodes under a node as a cluster. Note that singleton clusters (i.e., those with one labeled node) are trivial when all labeled nodes are placed at leaves; however, when labeled nodes can be placed at internal nodes, including or excluding singleton clusters can change the metrics. Thus, we also define many of the distances with and without singleton clusters. Some distances (i.e., FNR and FDR metrics) are already normalized. To normalize other distances, for each experimental condition, we create a control tree by randomly permuting labels of the true tree. We then normalize the errors of a reconstruction method by dividing it by the average score of replicates of the control method.

## 3 Results

### 3.1 Demonstration

**Figure 3:**
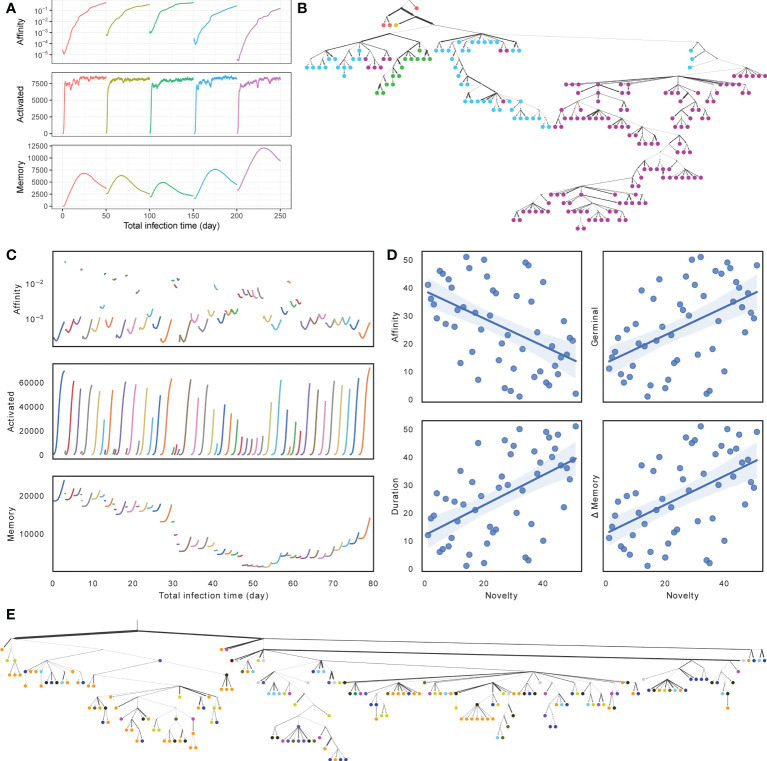
**(A, C)** Average affinity of activated cells to current infection target (log scale), the number of activated cells, and the number of memory cells by total time in infected stage across stages of infection (colors) for **(A)** SARS-Cov2 (discarding dormant stages) and **(C)** Flu (discarding the first 5 rounds and dormant stages). **(B, E)** Clonal tree of memory cells sampled from one simulation under default condition after all rounds for **(B)** SARS-Cov2 and **(E)** Flu. Nodes are colored by rounds when the memory cells emerge **(A, C)** (in Flu tree, gray color is used for rounds 1 through 46). Here, 11 internal nodes in SARS-CoV2 tree and 17 internal nodes in Flu tree are sampled and are indicated as circles. Edge weights denote the number of mutations of sequences denoted by adjacent nodes. **(D)** Impact of the novelty of the antigen on the outcome of the infection across the 56 rounds of influenza simulations. The novelty of rounds is measured by *−*max*_j<i_{*Δ*_ζi_* (*ζ_j_)}* and is ranked from less novel to more novel on the x axis. The y-axis shows ranking (from low to high) of average affinity of activated cells to the current infection target (*R* ^2^ = 0.242, *p* = 2.5 *×* 10^*−*4^) at the end of the infection, the number of activated cells (*R* ^2^ = 0.248, *p* = 2.0 *×* 10^*−*4^) at the end of the infection, the duration of infection (*R* ^2^ = 0.288, *p* = 4.8 *×* 10^*−*5^), and the change in memory cell count (*R* ^2^ = 0.264, *p* = 1.2 x 10^−4^) from the start to the end of the infection. See **Figure S1** for more details.

Visualizing one replicate under default conditions for both SARS-Cov2 and flu simulations, we see similarities and differences between the two scenarios (**Figures 3A, B** versus **Figures 3C–E**). In both cases, during each round of infection, the affinity first decreases and then increases as long as the duration of the infection is long enough (**Figures 3A, C**). Thus, when the number of activated cells is low, and the selective pressure is low, a mutation is likely to lead to reduced affinity, whereas when the number of activated cells increases, the selective pressure begins to increase and select for higher affinity; these patterns are in concordance with the literature (48). In the SARS-Cov2 simulations with a fixed duration, the total number of activated cells converges to similar values across infections, but the final affinity changes (**Figure 3A**). In the case of flu, the duration of infections, the mean affinity at the end, and the total number of cells vary widely across different rounds (**Figure 3C**). When the affinity at the start of a round is low, the duration of infection is longer, and more activated cells and memory cells are generated (**Figures 3C**, **S1A**). This pattern can be justified: when the immune system already has a high affinity to the antigen, it can eradicate the antigen quickly and without much need for further evolution. To further quantify the pattern, we define the novelty of each target *ζ_i_* as the negation of the maximum BLOSUM score between that target and any previous target: *−*max_*j<i*_{Δ*_ζi_* (*ζ_j_)*}. We observe that as novelty of the target increases, the average affinity of activated cells at the end of the infection tends to decrease (*R* ^2^ = 0.242, *p* = 2.5 *×* 10^*−*4^), whereas the number of activated cells at the end of the infection (*R* ^2^ = 0.248, *p* = 2.0 *×* 10^*−*4^) and the duration of infection (*R* ^2^ = 0.288, *p* = 4.8 *×* 10^*−*5^) both tend to increase (**Figure 3D**).

Memory cell counts fluctuate during each infection and across infections. In the SARS-Cov2 simulations, because the duration of the infection is fixed, after 15–30 days, the accumulation of memory cells stops; in fact, the buildup of memory cells starts to fade due to cell deaths, as one would expect. In the flu simulations, which have a much shorter duration, each round leads to a buildup in memory cells from the start to the end of the infection, and the amount of buildup depends on the duration and correlates with novelty (*R* ^2^ = 0.264, *p* = 1.2 *×* 10^*−*4^). However, the total number of memory cells reduces between rounds due to cell deaths (**Figure S1C**) and changes across rounds. In particular, a string of short-lived infections and large time spans between the flu seasons between 2002 and 2008 gradually lead to a depletion of the memory cells, which are then built up again in the subsequent rounds (**Figure S1C**). Finally, sequences from each round are not monophyletic in either simulation, as expected due to the use of memory of cells (**Figures 3B, E**). In both cases (especially SARS-Cov2), the antibodies evolved in later infections have more representations in the sample and are further away from the root.

### 3.2 Benchmarking methods

#### 3.2.1 SARS-Cov2 simulations

##### 3.2.1.1 Default parameters

**Figure 4:**
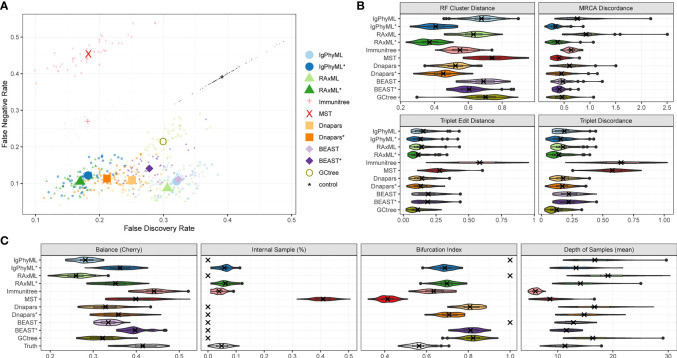
SARS-Cov2 simulations with default parameters. **(A)** False discovery rate (FDR) and false negative rate (FNR), **(B)** normalized Robinson-Foulds cluster distance (RF), MRCA discordance (MD), triplet edit distance (TED), and triplet discordance (TD) of various reconstruction methods all restricted to 48 replicates where all methods ran. The “control” trees are generated by randomly permuting labels of the true tree. Benchmarking results in **(B)** are normalized by the control trees. **(C)** Properties of the estimated and true trees. *: contracted trees.

Under default parameters, GCtree failed to run on 2 out of 50 replicates, and we excluded those replicates from our analyses. The relative accuracy of methods depends partially on the metric of choice (**Figure 4**). Using metrics that rely on cluster recovery (i.e., RF, FDR *vs*. FNR, and MD), the contracted phylogenetic ML methods (IgPhyML* and RAxML*) have the best accuracy. Judged by triplet-based metrics TED and TD, GCtree has the best accuracy (mean: 11% for TED and 12.4% for TD) followed closely by RAxML* (mean: 11.2% for TED and 14.1% for TD). While RAxML* is among the most accurate methods with all metrics, GCTree is very accurate according to triplet-based metrics but not with cluster-based methods. This discrepancy reflects different emphases of the metrics: TED and TD focus more on deeper branches in the tree that produce more triplets and are less sensitive to local branch contractions and resolutions. By contrast, cluster-based metrics treat every part of the tree equally and can be highly sensitive to local changes. The other methods have no obvious advantage to ML or GCtree. The MST-like methods have high precision, coming close to contracted ML methods, but also have much lower recall (FNR *>* 25%). Immunitree (which uses Steiner trees) is substantially better than a simple MST in terms of recall but not in terms of many metrics, including triplet-based measures (TED and TD). The MP method, Dnapars, was not more accurate than ML, and neither were BEAST or BEAST*.

Branch contraction improves the accuracy of all phylogenetic methods under all metrics (**Figure 4B**). Normal phylogenetic ML methods (IgPhyML and RAxML), which produce fully binary trees with all samples at leaves, have the lowest FNR (highest recall), retrieving approximately 90% of the correct clusters. However, their FDR is predictably high (low precision): more than 30% of their clusters are incorrect. Luckily, contracted ML methods have only a slight increase in FN rate (*<* 2% on average) but enjoy a dramatic improvement in precision. By simply contracting super-short branches, the FDR error reduces to less than 20% for IgPhyML* and RAxML*, which is better than all other methods. Similarly, normal phylogenetic methods perform poorly according to RF and MD metrics, which emphasize precision. Among the two phylogenetic ML methods, RAxML is slightly more accurate than IgPhyML in terms of RF, FNR, and FPR, while IgPhyML has slightly lower MD. The accuracy of MP and Bayesian methods also improve with branch contraction, but the improvements are less dramatic than ML. Dnapars* improves the precision of Dnapars by approximately 4% at the cost of only 0.4% decrease in recall. While BEAST has similar accuracy to RAxML before contraction, BEAST* is far inferior to RAxML* because it improves the precision over BEAST far less than RAxML* versus RAxML.

We next compare the properties of the inferred trees and true trees (**Figures 4C**, **S2**). MST puts far too many labels at internal nodes (*≈*40% instead of *≈*5%), while Immunitree and contracted ML trees are very close to the true tree in terms of percent internal samples. Immunitree overestimates the tree balance, while other methods underestimate balance, especially before contracting short branches. Conversely, MST and Immunitree underestimate and other methods overestimate the depth of samples; note that contraction also reduced error in sample depth. Phylogenetic methods, by definition, overestimate the bifurcation index as 1; this overestimation is dramatically reduced but not fully eliminated by contracted phylogenetic methods and Immunitree. MST, on the other hand, underestimates bifurcation index.

##### 3.2.1.2 Varying sample size

**Figure 5:**
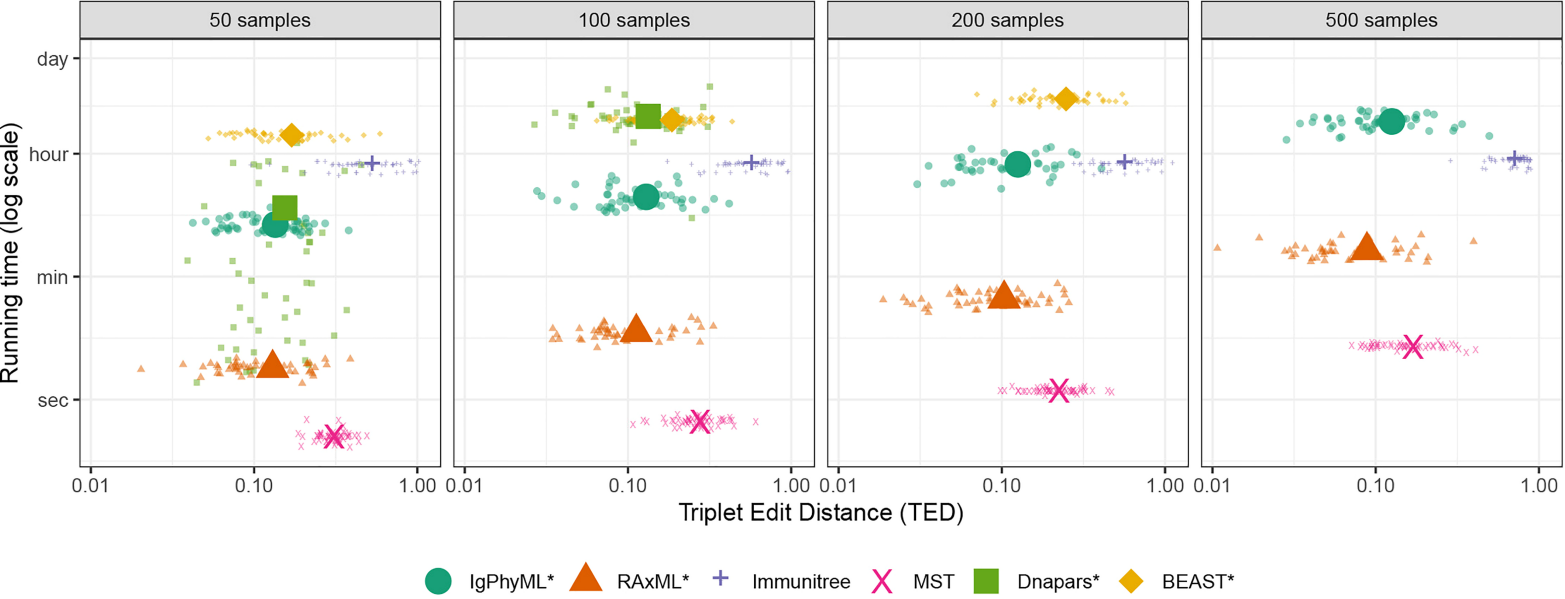
Running time and triplet edit distance (TED) of various reconstruction methods on SARS-CoV2 simulations under different sample sizes (50 replicates). Note that contracted methods have the same running time as non-contracted versions are hence are not shown. Also note that GCTree is not included but its running time increases similarly to Dnapare, which it runs internally.

As the number of sequences sampled changes from 50 to 500, the accuracy and running time both change (**Figures 5**, **S3**). The impact of sample size on the accuracy of IgPhyML* and Dnapars* measured according to TED is insignificant (*p* = 0.62, 0.18, respectively, according to an ANOVA test). Increasing sample size does significantly reduce the accuracy of Immunitree and BEAST* (*p* = 5 *×* 10^*−*6^ and *p* = 0.0001, respectively) and significantly improves the accuracy of RAxML*and MST (*p* = 0.006 and *p<* 10^*−*15^, respectively). The main impact of the sample size is on the running time. MST runs in seconds, and RAxML takes minutes to finish for all dataset sizes. All other methods were much slower. While Dnapars is the third fast method with 50-sequence input, its running time increases rapidly, and it fails to finish with *≥* 200 sample cases within 24 hours. In fact, Dnapars had the worst running time scaling. We empirically estimate that the running time of other methods increases with sample size no worse than quadratically (proportionally to *ς* ^1.5^, *ς* ^1.7^, *ς* ^0.07^, *ς* ^1.3^, and *ς* ^0.9^ for IgPhyML, RAxML, Immunitree, MST, and BEAST respectively) while Dnapars increases with *ς* ^7.4^ for the two data points where it did finish. Although we didn’t directly include GCtree, we can approximate the running time of GCtree by Dnapars, as invoking Dnapars is the most time-consuming step of GCtree. While BEAST scaled well, it had the highest running time and failed to finish 500 sample cases within the allotted 24 hours.

#### 3.2.2 Flu simulations

Because Dnapars, GCtree, and BEAST are much slower (and in the case of GCtree, occasionally fail to run properly), we do not include them for the flu analyses (where *ς* = 200).

##### 3.2.2.1 Default parameters

**Figure 6:**
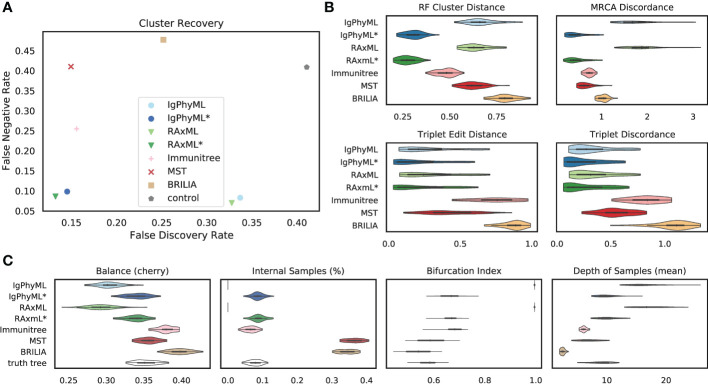
**(A)** False discovery rate (FDR) and false negative rate (FNR), **(B)** normalized Robinson- Foulds cluster distance (RF), MRCA discordance (MD), triplet edit distance (TED), and triplet discordance (TD) of various reconstruction methods on Flu simulations under default conditions (30 replicates). The “control” trees are generated by randomly permuting labels of the true tree. Benchmarking results in **(B)** are normalized by the control trees. **(C)** Properties of the estimated and true trees. For results excluding singletons and the PD metric, see **Figure S4**. *: contracted trees.

Under default parameters, Flu simulations exhibit very similar patterns to SARS-CoV2 simulations. Over all evaluation metrics, contracted ML methods (IgPhyML* and RAxML*) clearly have the best accuracy (**Figure 6**). By simply contracting super-short branches, the FDRs of IgPhyML and RAxML reduce to less than 15% from close to 35%. The MST-like methods still have low FDR and very high FNR. BRILIA consistently has a high error in our analyses. These patterns remain largely similar (but are magnified) when singletons are removed from consideration (**Figure S4**). The main exception is that when singletons are excluded, Immunitree is no longer the second-best method according to the RF distance. When comparing the properties of the inferred trees and true trees (**Figure 6C**), BRILIA puts far too many labels at internal nodes (*≈*35% instead of *≈*8%), overestimates the tree balance, underestimates the depth, and slightly underestimates the bifurcation index. On this dataset, MST is quite close to the correct levels of bifurcation.

##### 3.2.2.2 Varying selective pressure

**Figure 7:**
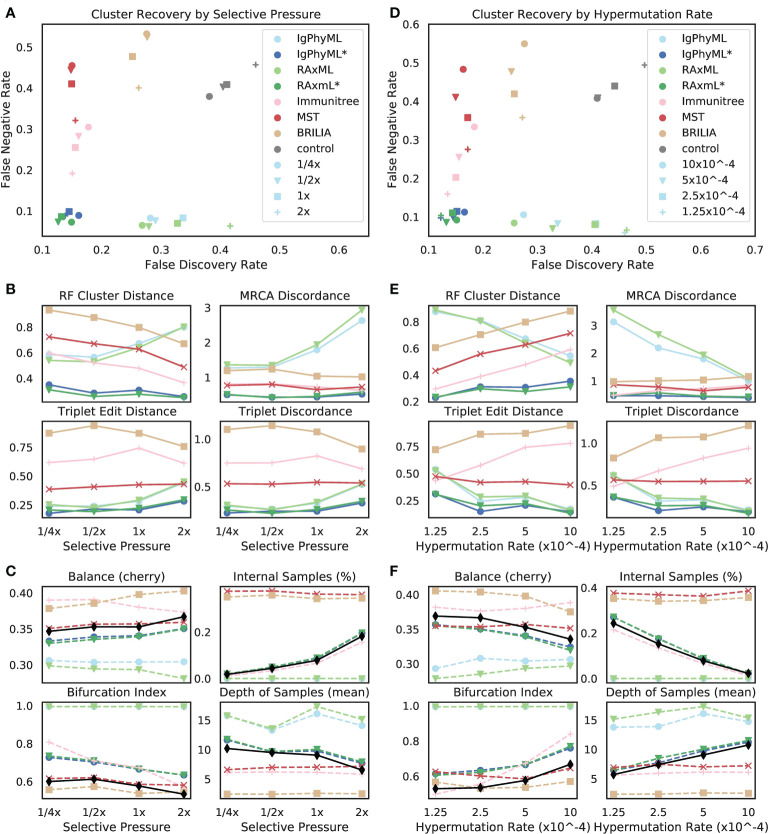
**(A-C)** Impact of selective pressure *A* and **(D-F)** mutation rate *µ* on **(A, B, D, E)** tree inference error and **(C, F)** tree properties. We measure tree error by **(A, D)** FDR, FNR, **(B, E)** Robinson-Foulds cluster distance (RF), MRCA discordance (MD), triplet edit distance (TED), and triplet discordance (TD). Tree errors and tree properties are averaged over 30 replicates. **(C, F)** We show properties of true (black) and reconstructed trees. *µ* = 5 *×* 10^*−*5^ in **(A-C)** and *A* = 0.1 in **(D-F)**, which are all default values. *: contracted trees.

The reconstruction methods are all impacted as selective pressure (*A*) changes, but some methods are more sensitive than others, and they are affected differently (**Figures 7A, B**). Contracted phylogenetic methods have the best accuracy across values of *A*. The ranking among other methods depends on the selective pressure, such that phylogenetic methods become the worst when *A* is high and become the best when *A* is low. As *A* increases, the error tends to increase for phylogenetic methods under all evaluation metrics except for the FNR; for example, the FDR of RAxML increases from 27% at the 1*/*4x selective pressure to 42% at the 2x level. In contrast, the error of Immunitree, MST, and BRILIA reduces with increased *A* according to FNR and RF. Contracted phylogenetic methods are relatively robust to the *A* and their error rates change only slightly across conditions. When singletons are removed from the metrics of comparison, patterns remain similar, though the impact of selective pressure becomes less pronounced (**Figure S5A**).

The reason behind these patterns becomes more apparent once we consider changes in tree properties (**Figure 7C**). As *A* increases, the fraction of internal samples tends to increase. This pattern can be explained: when selective pressure is high, cells with low affinity die off quickly, which results in shorter branch lengths. Since phylogenetic methods put all sequences at leaves, they have reduced accuracy. In contrast, IgPhyML*, RAxML*, and Immunitree successfully assign sequences to internal branches; as a result, their percentages of internal samples match those of the true trees. Similarly, with increased *A*, the bifurcation index of the simulated tree tends to decrease, a pattern that is observed also in reconstructed trees from IgPhyML*, RAxML*, Immunitree, MST, and BRILIA. Again, phylogenetic trees, which produce binary trees, are unable to capture these patterns. As *A* increases, the depth of sampled nodes of the simulated tree tends to decrease, a pattern matched by IgPhyML* and RAxML* but not other methods. Finally, when *A* is high, trees are shorter (i.e., accumulate fewer mutations) and more branches are single mutation (**Figure S6**), both of which make phylogenetic inference more difficult. The reduced levels of depth, total change, and bifurcation make sense: higher pressure should result in fewer mutations needed to reach *M* because cells with unfavorable mutations are less likely to survive; this would produce shorter trees.

##### 3.2.2.3 Varying rate of hypermutation

As the hypermutation rate (*µ*) increases, error decreases for normal phylogenetic methods (Ig- PhyML and RAxML) according to most metrics but stays relatively stable for contracted methods (**Figures 7D, E**). Increasing *µ* results in simulated trees that are marginally less balanced, are more bifurcating, have fewer internal node samples, and have a higher depth for sampled nodes (**Figure 7F**). Thus, increasing *µ* generates trees more similar to what traditional phylogenetic methods assume. Contracted phylogenetic methods and Immunitree designate the right percentage of nodes as internal, but both are slightly more bifurcating than true trees (**Figure 7F**). Overall, contracted phylogenetic methods are the most accurate across all values of *µ*.

##### 3.2.2.4 Interplay between selective pressure and mutation rate

**Figure 8:**
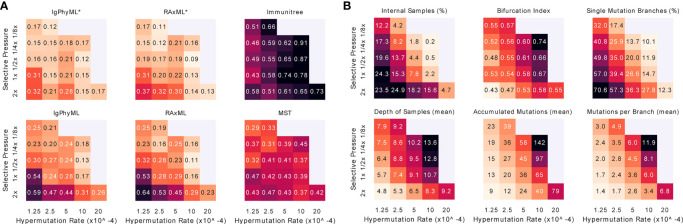
For varying levels of selective pressure **(A)**, rate of hypermutation (*µ*), and all reconstruction methods except BRILIA, we show **(A)** tree error measured by the triplet edit distance TED and **(B)** properties of the true tree. When the mutation rate is too high and the selective pressure is too low, the simulation never ends, meaning that the total affinity needed to overcome the antigen is never reached; these conditions are missing from the figure. For other evaluation criteria see **Figure S7**. *: contracted trees.

When we vary both *A* and *µ*, we observe that increasing the mutation rate has similar effects on the error and tree properties as decreasing the selective pressure (**Figure 8**). Reassuringly, error patterns observed when fixing one variable and changing the other are consistent with patterns when both variables are changed (**Figures 8**, **S7**). The most difficult condition for phylogenetic methods is low mutation rates and high selective pressure, where close to 70% of the branches include only a single mutation, and the bifurcation index is only 43%. However, the contracted methods are impacted less in these conditions and are, in fact, improved according to the RF metric (**Figure S7**). In addition, we observe that antibody clonal trees become more phylogenetic-like – that is, more bifurcating (max: 0.74) and fewer internal samples (min: 20%) – with *µ* = 10^*−*3^ and *A* = 1*/*4x. Increasing the mutation rate or decreasing the selective pressure beyond these values leads to combinations where the infection could not be overcome.

##### 3.2.2.5 Other parameters

**Figure 9:**
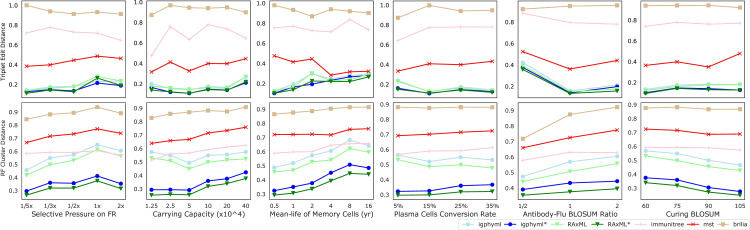
Triplet edit distances and RF cluster distances by selective pressure on framework region, carrying capacity, mean-life of memory cells, plasma cell conversion rate, antibody-flu BLOSUM ratio (MARatio), stage change threshold (M).

Beyond the main two parameters, we also studied changing six secondary parameters, most of which had relatively little impact on the results (**Figure 9**). As the weight of FRs regions in computing affinity (*w_f_)* increases, the error tends to increase slightly for all methods under many evaluation metrics (**Figure S8**). This pattern can be related to the slight increase in the number of single branch mutations and the reduction in the total number of substitutions across the tree. As germinal center capacity (*C*) increases, error increases or decreases slightly, depending on what measure is examined (**Figure S9**). Increasing *C* tends to reduce the number of internal samples and single mutation branches in the simulated tree, and tends to increase mutations per branch. As memory cell life-time (1/ $\lambda_{d}^{'}$ ) increases, the error tends to increase for phylogenetic methods (**Figure S10**), including IgPhyML* and RAxML*, which nevertheless continue to be the best methods. Plasma cells conversion rate (*ρ_p_*) (**Figure S11**), rate of change in antibody target compared to antigen change (*κ*) (**Figure S12**), and the threshold of total affinity for neutralization and stage change (*M*) (**Figure S13**) have small and inconsistent impacts on tree inference error. In all conditions examined, IgPhyML* and RAxML* have the best accuracy (**Figure 9**).

## 4 Discussion

We introduced a general model of antibody evolution and instantiated it in two different ways to simulate antibody response to flu and SARS-Cov2. The two simulations were substantially different in their choice of models and model parameters and produced quite distinct scenarios (**Figure 3**). Nevertheless, they showed similar results in terms of benchmarking of methods. Below, we further comment on the implications of these results for reconstruction methods, evaluation metrics, and simulation procedures. We end by pointing out the limitations of the current work and directions for future work.

### 4.1 Implications for reconstructing antibody evolution

Consistent with the literature, we found that ML methods need subsequent processing for inferring antibody clonal trees with high accuracy. Depending on the simulation condition, 1% to 20% of sampled sequences belonged to internal nodes, and the true trees are only 60% to 70% bifurcating. We observed that results of phylogenetic inference using ML, taken at face value, can have low accuracy. However, ML phylogenetic methods with the simple adjustment of contracting short branches can outperform the alternative methods. Despite the higher accuracy of contracted phylogenetic methods compared to the available alternatives, we note that there is still substantial error. Under the default condition, while 90% of clusters of the true tree were recovered, about 15% of the recovered clusters were incorrect. In particular, the discrepancy between FNR and FDR was due to the fact that the inferred trees are somewhat more bifurcating than true trees (e.g., *≈*70% versus 60% in the default condition). Thus, while contracting super-short branches increases accuracy, these trees are still biased towards too much resolution.

Previous benchmarking of reconstruction methods has shown somewhat contradictory results in terms of the choice of the best methods. Davidsen and Matsen (32) found parsimony-based methods GCTree and another post-processing method they introduced called SAMM to be superior to ML methods (they did not test Bayesian methods). In contrast, Yermanos et al. (49) concluded the Bayesian method BEAST is the best method overall, followed by ML and MP methods. The two studies used very different simulation approaches. In addition, Davidsen and Matsen (32) used the genotype-collapsing approach adopted from DeWitt et al. (26) to create contracted trees, while Yermanos et al. (49) did not. Our study, which has more similarities to Davidsen and Matsen (32) than Yermanos et al. (49), only partially confirms observations from these previous studies.

Just like Davidsen and Matsen (32), we observed that subsequent processing improves MP methods, as GCTree was better than Dnapars in our tests. Similar to them, we also ranked GCTree highly when judged by triplet-based distances. However, Davidsen and Matsen (32), who used dnaml and IQ-TREE, found that ML methods are inferior to MP methods. Our results ranked RAxML* (RAxML plus contraction) as the best or close to the best method in all simulations with all metrics. The exact causes of these disparities are not obvious. They could stem from differences in simulation procedures, evaluation metrics used (recall that with triplet metrics, GCtree is slightly better than RAxML*), or even the choice of the ML inference method (dnaml and IQ-TREE versus RAxML). More consequentially, we used the Γ model of rate-across-sites heterogeneity, but it appears that Davidsen and Matsen (32) ran IQ-TREE with no model of rate heterogeneity, which may be problematic given that antibody sequences include hotspots and coldspots. A final interesting difference is the contraction strategy. In contrast to Davidsen and Matsen (32) who used ancestral reconstruction for contracting branches, we used a fixed branch length threshold that we selected based on the sequence length. It could be that difficulties in ancestral state reconstruction render the genotype-collapsing approach of DeWitt et al. (26) less effective than our simple rule of thumb. Note that statistical tests of whether a zero branch length null hypothesis can be rejected also exist (50–52) and are fast (53) and could be used in lieu of our simple heuristic, an option that should be tested in the future. Our results imply that phylogenetic methods that naturally model zero branch length [e.g., (54)] are also promising. In particular, the adaptive LASSO method of Zhang et al. (55) seems suitable for inferring antibody evolution and can be tested in the future.

It is harder to reconcile our results with Yermanos et al. (49). Unlike their study, our results did not find BEAST to be better (or much worse) than ML method RAxML. The main limitation of BEAST in our analyses was that it did not benefit from branch contraction nearly as much as ML methods do. This difference is likely because the prior distributions on branch length render our simple rule-of-thumb ineffective. For ML methods, branches with lengths much smaller than the inverse of the sequence length can be expected to correspond to no changes. This simple rule fails when the branch lengths is governed by priors as well as the likelihood. It is possible that Bayesian polytomy tests (54) could overcome this issue and produce accurate results. It is also likely that with different priors on branch length, our simple heuristic could become more effective. More broadly, the topological accuracy of BEAST may be impacted by priors (we attempted to use settings similar to Yermanos et al. (49), but other priors may increase accuracy). These uncertainties are not limited to our study and point to a practical difficulty with using Bayesian MCMC methods: given the limited signal in antibody sequences, the prior choice is consequential and needs more research.

Beyond accuracy, methods had substantially different running times in ways that may seem surprising. As expected, BEAST was slower than other methods, though its running time rose only linearly as the datasets became larger. However, RAxML was dramatically faster than the MP method Dnapars. While MP is a somewhat simpler problem than ML, the available tools for MP inference are not as advanced as ML methods. As a result, we did not see a trade-off between running time and accuracy; except for MST, which was extremely fast but inaccurate, RAxML* had the best running time and the best accuracy according to most criteria. Larger samples can give a more complete picture of the evolutionary changes. Nevertheless, accuracy was not impacted dramatically by sampling (except for MST), and only 50 samples were enough to obtain reasonable accuracy, indicating that high sampling may not be always essential.

### 4.2 Implications for evaluation criteria

The ranking of reconstruction methods can change based on which of the ten evaluation criteria we choose, and these rankings only partially correlate (**Figure S14**). FDR and FNR are weakly anti-correlated only when including singletons (mean Spearman’s rank correlation coefficient across all tests *ρ* = *−*0.12). RF distance, which combines both aspects, correlates moderately with both FDR (*ρ* = 0.5) and FNR (*ρ* = 0.57). The triplet-based metrics strongly agree with each other (*ρ* = 0.97) and are mostly compatible with the RF distance (*ρ ≈* 0.75), but are less similar to MD and PD metrics (*ρ ≤* 0.52). Consistent with the observation that triplet metrics penalize false negatives more than false positives, they agree more strongly with FNR than FDR (*ρ* = 0.65 *vs* 0.26). MD and PD are very similar to each other (*ρ* = 0.96), have no correlation to FNR (*ρ ≤* 0.05), but have moderately high correlation to FDR (*ρ* = 0.71). Finally, we notice that singletons can matter: while FNR and FNR* are highly correlated (*ρ* = 0.94), RF correlates with RF* less strongly (*ρ* = 0.71), and FDR correlates with FDR* only moderately l (*ρ* = 0.61).

The choice of the metric should depend on the downstream application of the clonal tree. While contracted phylogenetic methods are dramatically better than base methods based on most criteria, they are only slightly better according to the triplet-based criteria. The triplet metrics do not penalize trees heavily if they are more resolved than the true tree or if they move internal nodes to leaves. Thus, when downstream usage is robust to extra resolution and extra terminal edges, triplet metrics offer a good way to measure topological accuracy. On the other extreme, PD and MD are very sensitive to the tree resolution and internal placement, so much so that they often evaluate inferred phylogenetic trees to be much worse than random trees (**Figure S7**) because these trees generate fully resolved trees and put samples at leaves. Thus, we don’t find PD and MD to be reliable metrics of topological accuracy. RF distance is in between: it penalizes extra resolution more than triplet metrics but less than path-based metrics. It does distinguish contracted and phylogenetic methods but rarely evaluates any methods to be worse than random (**Figure S7**). Overall, dividing the observed error along two (potentially contradictory) axes, such as FNR and FDR is recommended because this evaluation provides more insight into the reasons behind errors.

### 4.3 Comparison to other simulation models

Several simulation tools capable of benchmarking reconstruction methods have been developed. Some of these tools are not comparable to our effort because of various limitations. ImmuneSIM (56) generates mutations but does not model the clonal tree or the selection process. Methods of Amitai et al. (30) and Reshetova et al. (31) are based on the two-step simulation paradigm and only generate clonal trees under selection, leaving sequence generation to other methods. AbSim (49) models VDJ recombination and does not model memory cells; moreover, it appears that the tree shape evolves independently from sequences. The most relevant method to ours are bcr-phylo (32) and gcdynamics (29), which simulate clonal trees of antibody-coding sequences under AM. Both bcr-phylo and gcdynamics have similarities and differences to our method (**Table 5**). For example, they both support multiple targets but only one round of simulations. Although our model is capable of multiple targets, for simplicity, DIMSIM uses one target per round of infection. However, unlike the two other methods that only simulate activated cells, DIMSIM also simulates memory cells; as a result, it can simulate multiple rounds of infection by an evolving pathogen with changing targets while considering memory built from previous infections. Moreover, DIMSIM simulates in continuous time, whereas the other tools simulate under discrete generations. All three methods use sequences to define affinity, albeit differently: DIMSIM using BLOSUM distance, brc-phylo using hamming distance, and gcdynamics using random energy landscape. A main feature of DIMSIM is that its rates are polynomial fractions of individual and total affinity; this choice enables it to speed up the simulation, allowing it to scale up to large numbers of cells.

**Table 5:** A comparison of Most relevant tools for AM simulation.

	DIMSIM paper	bcr-phylo (32)	gcdynamics this (29)
Targets	Single-target (per round)	Multi-target (1 round)	Multi-target (1 round)
Rounds	Yes	No	No
Affinity	BLOSUM distance	Hamming distance	Random energy landscape
Mutation	Updated (39)	(39)	i.i.d
Scalability	Up to millions of cells	Thousands of cells	Thousands of cells
Cell type	Activated and Memory	Activated	Activated
Germinal Centers	Combined (single)	Combined (single)	Multiple (in competition)
Time	Continuous	Discrete generations	Discrete generations
Isotype	No	Yes	No
Birth/Death rate	Polynomial fraction of individual and total affinity	Neutral: independent of total affinity Kinetic: function of affinities	A function of affinity

### 4.4 Limitations of the study

Our study has limitations that should be kept in mind. While many of these limitations may impact the realism of the model, most will likely not impact the relative accuracy of reconstruction methods.

In our simulations, we did not add errors to sequence data used as input to clonal tree reconstruction methods. Real AIRR-seq samples undergo extensive PCR and thus might contain both sequencing and amplification errors. We assumed that error elimination is already performed (to perfection) prior to reconstruction using existing methods [e.g., (57–60)]. The efficacy of methods that simultaneously filter errors and build clonal trees [e.g., (22, 61)] should be the subject of future research. We also simulated only substitution SHMs but no insertions and deletions, leaving the latter to future work.

In our AM model, we made several simplifying assumptions. For example, we assumed the affinity grows gradually as the AA sequence becomes more similar to the target sequence. The idea that AM occurs by mutational diffusion along one or more preferred paths in the genotype space has been supported by Kepler et al. (62). Nevertheless, our i.i.d model is certainly a simplification without clear empirical support. More complex models of receptor binding exist and can be integrated in the future: Ymir (63) is a 3D structural affinity model which takes into account affinity jumps, cross-reactivity, and differential epitope accessibility. There are also machine learning attempts (64) to build predictive models of affinity, which can be incorporated. However, including such sophisticated models could drastically slow down simulations.

We also assumed the existence of a target antibody sequence. The literature has increasingly documented highly convergent immune responses to the same epitope across individuals and conditions (65, 66). This observation gives us reason to think the existence of target sequences is not a bad assumption; nevertheless, the choice of a single target may not be realistic. To model the change in the target as the viruses evolve, we used two approaches: in one, we used real antibody sequences, which seems like a reliable approach. In our second experiment, we chose targets with evolutionary divergence levels that mimic the divergence levels of the antigen, albeit with some scaling factor. In the latter case, it is conceivable that two antigens with high evolutionary distance are neutralized by similar antibodies or that antigens that are very similar require very distant antibodies. We modeled SHMs as affecting daughter cells independently, but it is arguably more realistic to make both daughter cells carry the same mutation due to the G1 origin of SHMs (37) (a simple change to the model). Finally, our 5-mer mutation model, while based on the empirical model of Yaari et al. (39), still fails to capture some complexities of the real antibody evolution. For example, we concentrated substitutions on the CDR region, but other regions are known to also accumulate mutations (61, 67, 68). Other B cell specific models [e.g., (69)] including those that seek to tease out the effects of selection from background mutations [e.g., (70)] and per-position mutability models (62) can be incorporated in the future.

For all these shortcomings, we offer several responses. Due to challenges in modeling antibody repertoire [e.g., (71)], the framework is designed to be flexible and can easily incorporate more complex models. Thus, our work should be considered a first step that will enable better modeling in the future. Also, our objective in simulations was to benchmark reconstruction tools; as long as our modeling choices did not distort the comparison of methods, some model misspecification can be tolerated. We observed that the choice of the best method was not sensitive to many parameter choices, and in fact, the two simulations resulted in similar conclusions.

Beyond model simplifications, we also chose to simulate parts of the complex immune system response but not others. For example, we simulated one clonal lineage involved in an immune response. As such, we ignored the important V(D)J recombination of IG loci (72) and sought to simply simulate a VDJ recombinant that is effective in fighting a specific antigen. Even then, we simulated only one clonal lineage at a time, a limitation that can be easily lifted in the future by starting from multiple root sequences with different VDJ settings and assigning to each a different target sequence. Note that our tool can be easily combined with methods of simulating VDJ recombination, such as IGoR (73). Neither did we simulate light chains, which are often not captured in AIRR-seq sequencing data. Finally, we did not simulate processes such as epitope focusing that produce broadly neutralizing antibodies (74).

### 4.5 Applications of the framework

Our framework for simulating clonal trees can be extended to other forms of microevolutionary scenarios. While the current implementation is geared towards AM simulations, our proposed algorithm enables forward-time simulation of very large numbers of entities under models that allow dependence between sequences and rates of birth, death, or transformation. The ability to simulate a very large number of entities combined with rates that change with properties of entities gives us the necessary ingredients to simulate under complex models of evolution that consider selective pressure. Thus, our framework can be adopted for other forms of microevolutionary simulation, such as the evolution of a virus within a host and accumulation of SHMs in tumor evolution. Such a possibility would become most intriguing if it can also model the co-evolution of different types of entities (e.g., antibodies and viruses). While we did not simulate co-evolution here, we believe the framework is capable of performing such simulations by simply creating entity types (just like we had cell types) and making the BDT rates a function of properties across different cell types. Another promising direction for extensions of this work is to integrate the sequence evolutionary models with network-based disease transmission models [e.g., (75, 76)] to enable more accurate simulations of disease spread and evolution.

## Data availability statement

The original contributions presented in the study are included in the article/**Supplementary Material**. Further inquiries can be directed to the corresponding author.

## Author contributions

CZ designed and implemented the simulation algorithm. AB generated the empirical 5-mer mutation model. CZ, YS, and SM designed the experiments. CZ and SM designed evaluation metrics for banchmarking. CZ and SM analyzed the results. All authors contributed to the article and approved the submitted version.

## Funding

This work was supported by National Science Foundation (NSF) grant III-1845967 and National Institute of health (NIH) grant R35GM142725. Computations were performed on the San Diego Supercomputer Center (SDSC) through XSEDE allocations, which is supported by the NSF grant ACI-1053575.

## Acknowledgments

We would like to thank Pavel A. Pevzner, Li-Fan Lu, and Jiawang Nie for insightful discussions on clonal tree reconstruction, affinity maturation, and optimization methods. We would also like to thank Yuan Wang for professional figure editing.

## Conflict of interest

The authors declare that the research was conducted in the absence of any commercial or financial relationships that could be construed as a potential conflict of interest.

## Publisher’s note

All claims expressed in this article are solely those of the authors and do not necessarily represent those of their affiliated organizations, or those of the publisher, the editors and the reviewers. Any product that may be evaluated in this article, or claim that may be made by its manufacturer, is not guaranteed or endorsed by the publisher.
