# Supplementary material for: A scalable model for simulating multi-round antibody evolution and benchmarking of clonal tree reconstruction methods
Source: Front Immunol. 2022 Dec 6;13:1014439. doi: 10.3389/fimmu.2022.1014439 (PMC9815712; doi:10.3389/fimmu.2022.1014439)
Supplement: Supplementary file 1 [file DataSheet_1.pdf]

Supplementary Material

1 BRIEF INTRODUCTION OF RELEVANT CONCEPTS

Antibodies.

Antibodies are produced by *B cells* and are used by the immune system to recognize, bind, and neutralize pathogens. Antibodies are proteins consisting of immunoglobulin (IG) molecules of identical heavy chains and identical light chains. Immunoglobulins are encoded by B-cell receptor (BCR) sequences. Unlike other proteins, IGs are not encoded in the genome directly but present results of somatic *V(D)J recombination* of *IG loci* (Kurosawa and Tonegawa, 1982). Each chain of each IG is encoded by a concatenation of one of V, D (only for heavy chain), and J genes, known as an *IG gene*. An IG gene contains three *complementarity-determining regions* (CDRs) representing antigen binding sites. CDRs are separated by four *framework regions* (FRs) that form a stable structure displaying CDRs on the antibody surface.

AM process.

After successful binding of an IG to a given pathogen, the corresponding B cell undergoes the *affinity maturation* (AM) process aiming to improve its *affinity* (i.e., binding ability) to the antibody (Tonegawa, 1983; Neuberger and Milstein, 1995). First, the targeting B cell moves to a *germinal center* (GC) of a lymph node, where it undergoes *clonal expansion*: cell divisions that increase the pool of antibodies that bind to the antigen. Then, certain enzymes in the B cell and its clones are activated and introduce *somatic hypermutations* (SHMs) in the utilized IG genes as a means to improve affinity (Muramatsu et al., 2000). SHMs change the three-dimensional structure of an antibody (and thus its ability to bind to an antigen) stochastically. The regulatory mechanisms of the immune system play the role of natural selection by expanding B cells with high affinity for antigen and killing self-reactive B cells with potentially harmful mutations. The AM process activates naive B cells (i.e., those that have not been exposed to an antigen) and differentiates them into *memory* and *plasma* B cells. Memory B cells can be repeatedly activated and subjected to the AM Mesin et al. (2020), while plasma B cells can secrete massive levels of neutralizing antibodies. Studies show that CDRs, which include the binding sites, accumulate more SHMs compared to FRs (Hsiao et al., 2019; Safonova and Pevzner, 2019).

Clonal expansion.

The AM process leads to the formation of clonal lineages within a given antibody repertoire, where each clonal lineage is formed by descendants of a single naive B cell. The expressed IG transcripts within the same clonal lineage share a common combination of V, D, and J genes and differ by SHMs only. The evolutionary history of each clonal lineage can be represented by a *clonal tree*, where each vertex corresponds to a B cell and each B cell is connected by a directed edge with all its immediate descendants.

2 SUPPLEMENTARY METHODS

2.1 Efficient sampling from the BDT model

Recall that because of the memoryless property, the time until the next BDT event always follows the exponential distribution with rates $\Lambda_B(\mathbf{x}_i, \mathbf{S}), \Lambda_D(\mathbf{x}_i, \mathbf{S})$, and $\Lambda_T(\mathbf{x}_i, \mathbf{S})$ for each event type. The time until *any* event for *any* entity follows an exponential distribution with rate

$$\lambda = \sum_{i \in S} (\Lambda_B(\mathbf{x}_i, \mathbf{S}) + \Lambda_D(\mathbf{x}_i, \mathbf{S}) + \Lambda_T(\mathbf{x}_i, \mathbf{S})) \ .$$

The probability of the next event being a specific event $E \in \{B, D, T\}$ for a particular entity i is

$$\frac{\Lambda_E(\mathbf{x}_i, \mathbf{S})}{\lambda} \ .$$

We **assume** that we are able to write

$$\Lambda_E(\mathbf{x}_i, \mathbf{S}) = \frac{P_E(\mathbf{x}_i, \mathbf{S})}{Q(\mathbf{S})}$$

where $P_E : \mathbb{R}^N_{\geq 0} \times \mathbb{R}^N_{\geq 0} \to \mathbb{R}_{\geq 0}$ and $Q : \mathbb{R}^N_{\geq 0} \to \mathbb{R}_{>0}$ are polynomial functions with a constant degree, where coefficients of P_E are non-negative. With this assumption, for any entity $i \in S$, the birth rate can be written as

$$\Lambda_B(\mathbf{x}_i, \mathbf{S}) = \frac{\sum_{\alpha, \beta \in \mathbf{\Gamma}} \mathcal{B}_{\alpha,\beta} \mathbf{S}^\beta \mathbf{x}_i^\alpha}{\sum_{\beta \in \mathbf{\Gamma}} Q_\beta \mathbf{S}^\beta}$$

where $\mathbf{\Gamma} = [0 \dots \gamma]^N$ for some integer γ, $\mathcal{B}_{\alpha,\beta}$ and Q_β are coefficients of the polynomials, and $\mathbf{a}^\mathbf{b}$ denotes $\prod_i \mathbf{a}_i^{\mathbf{b}_i}$ for vectors $\mathbf{a}$ and $\mathbf{b}$. We can write $\Lambda_D(\mathbf{x}_i, \mathbf{S})$ and $\Lambda_T(\mathbf{x}_i, \mathbf{S})$ similarly by replacing $\mathcal{B}_{\alpha,\beta}$ with $\mathcal{D}_{\alpha,\beta}$ and $\mathcal{T}_{\alpha,\beta}$. Note that in our specific AM model, rates shown in Table S1 follow this assumption.

With this assumption, we can write

$$\lambda = \frac{\sum_{\alpha, \beta \in \mathbf{\Gamma}} P_{\alpha,\beta} \mathbf{S}^\beta \theta_\alpha}{\sum_{\beta \in \mathbf{\Gamma}} Q_\beta \mathbf{S}^\beta}$$

where $P_{\alpha,\beta} = \mathcal{B}_{\alpha,\beta} + \mathcal{D}_{\alpha,\beta} + \mathcal{T}_{\alpha,\beta}$ and $\theta_\alpha = \sum_{i \in S} \mathbf{x}_i^\alpha$ for all α values (note that $\mathbf{S} = \theta_1$). Thus, to efficiently sample the time till the next event, we only need θ_α values which we can simply store and update in constant time after each event. This fast storing and updating allows for a constant

Table S1. Birth, death, and transformation rate functions as polynomials.

Rate functions	Infected stage	Dormant stage
$\Lambda_B(\mathbf{x}_i, \mathbf{S})$	$\lambda_b g_i$	0
$\Lambda_D(\mathbf{x}_i, \mathbf{S})$	$\frac{\lambda_b(1-\rho_p-\rho_m)}{C}(\frac{g_i}{a_i})\sigma + (\rho_p\lambda_b - \lambda'_d)g_i + \lambda'_d$	$(\lambda_d - \lambda'_d)g_i + \lambda'_d$
$\Lambda_T(\mathbf{x}_i, \mathbf{S})$	t_i	0

time sampling of the next event time (in terms of n) for constants N and γ. Once we sample the time till the next event, we need to sample one of the three possible events. The probability of the next event being birth for an entity i is

$$\begin{aligned}
\frac{\Lambda_B(\mathbf{x}_i,\mathbf{S})}{\lambda} &= \frac{\Lambda_B(\mathbf{x}_i,\mathbf{S})}{\sum_{j\in S}(\Lambda_B(\mathbf{x}_j,\mathbf{S})+\Lambda_D(\mathbf{x}_j,\mathbf{S})+\Lambda_T(\mathbf{x}_j,\mathbf{S}))} \\
&= \frac{\sum_{\alpha,\beta\in\mathbf{\Gamma}}\mathcal{B}_{\alpha,\beta}\mathbf{S}^{\beta}\mathbf{x}_i^{\alpha}}{\sum_{\alpha,\beta\in\mathbf{\Gamma}}P_{\alpha,\beta}\mathbf{S}^{\beta}\theta_{\alpha}} = \sum_{\alpha,\beta\in\mathbf{\Gamma}}\Big(\mathcal{B}_{\alpha,\beta}\mathbf{S}^{\beta}\mathbf{x}_i^{\alpha}\frac{1}{\sum_{\bar{\alpha},\bar{\beta}\in\mathbf{\Gamma}}P_{\bar{\alpha},\bar{\beta}}\mathbf{S}^{\bar{\beta}}\theta_{\bar{\alpha}}}\Big) \\
&= \sum_{\alpha,\beta\in\mathbf{\Gamma}}\Big((\frac{\mathcal{B}_{\alpha,\beta}\mathbf{S}^{\beta}\mathbf{x}_i^{\alpha}}{P_{\alpha,\beta}\mathbf{S}^{\beta}\theta_{\alpha}})(\frac{P_{\alpha,\beta}\mathbf{S}^{\beta}\theta_{\alpha}}{\sum_{\bar{\alpha},\bar{\beta}\in\mathbf{\Gamma}}P_{\bar{\alpha},\bar{\beta}}\mathbf{S}^{\bar{\beta}}\theta_{\bar{\alpha}}})\Big) \\
&= \sum_{\alpha,\beta\in\mathbf{\Gamma}}\Big((\frac{\mathcal{B}_{\alpha,\beta}}{P_{\alpha,\beta}})(\frac{\mathbf{x}_i^{\alpha}}{\theta_{\alpha}})(\frac{P_{\alpha,\beta}\mathbf{S}^{\beta}\theta_{\alpha}}{\sum_{\bar{\alpha},\bar{\beta}\in\mathbf{\Gamma}}P_{\bar{\alpha},\bar{\beta}}\mathbf{S}^{\bar{\beta}}\theta_{\bar{\alpha}}})\Big) \ .
\end{aligned} \tag{S1}$$

Also note that probability of each death and transformation event can be written similarly. This equation enables an efficient sampling procedure detailed in Algorithm S1 of Appendix 4:

1. Sample (α,β) pair (representing one term of the polynomial) from a multinomial distribution on $\mathbf{\Gamma}\times\mathbf{\Gamma}$ where each pair has probability $\frac{P_{\alpha,\beta}\mathbf{S}^{\beta}\theta_{\alpha}}{\sum_{\bar{\alpha},\bar{\beta}\in\mathbf{\Gamma}}P_{\bar{\alpha},\bar{\beta}}\mathbf{S}^{\bar{\beta}}\theta_{\bar{\alpha}}}$.
2. Sample entity i from a distribution on S where each i has probability $\mathbf{x}_i^{\alpha}/\theta_{\alpha}$.
3. Sample birth, death, or transformation with probabilities $\frac{\mathcal{B}_{\alpha,\beta}}{P_{\alpha,\beta}}$, $\frac{\mathcal{D}_{\alpha,\beta}}{P_{\alpha,\beta}}$, and $\frac{\mathcal{T}_{\alpha,\beta}}{P_{\alpha,\beta}}$.

In this procedure, the probability of selecting the birth event for an entity i is simply $\sum_{\alpha,\beta}\frac{\mathcal{B}_{\alpha,\beta}}{P_{\alpha,\beta}}\frac{\mathbf{x}_i^{\alpha}}{\theta_{\alpha}}\frac{P_{\alpha,\beta}\mathbf{S}^{\beta}\theta_{\alpha}}{\sum_{\bar{\alpha},\bar{\beta}\in\mathbf{\Gamma}}P_{\bar{\alpha},\bar{\beta}}\mathbf{S}^{\bar{\beta}}\theta_{\bar{\alpha}}}$, which matches Equation (S1) (ditto for death and transformation events). In terms of running time:

1. Step 1 takes constant time (in terms of n) given that θ_{α} values (and thus $\mathbf{S}$) are pre-computed for all α.
2. Step 2 can be achieved in $O(\log n)$ time using an interval tree data structure to store partial sums of $\mathbf{x}_j^{\alpha}$'s (see Algorithm S1).
3. Step 3 takes constant time.

Thus, a tree on k nodes drawn from the distribution defined by the BDT process can be sampled in $O(k\log(k))$ time by repeated applications of Algorithm S1.

2.2 Somatic hypermutagenesis frequency models

We next show the model for $\mathbf{K}^5$ and f. Our model is based on an empirical frequency $\mathbf{K}^5(s, s_1, s_2, s_3, s_4, s_5)$ matrix that counts the number of times 5-mer $(s_1, s_2, s_3, s_4, s_5)$ converts to (s_1, s_2, s, s_4, s_5) in one cycle of cell division during hypermutation. Given the matrix, we define

$$f(s, s_1, s_2, s_3, s_4, s_5) = \begin{cases} \mathbf{K}^5(s, s_1, s_2, s_3, s_4, s_5)\frac{\mu}{\text{RateEmp}} & s \neq s_3 \\ 1 - \sum_{s' \in \{A,C,G,T\}-\{s\}} \mathbf{K}^5(s', s_1, s_2, s_3, s_4, s_5) & s = s_3 \end{cases} \quad \text{(S2)}$$

where

$$\text{RateEmp} = 1 - \frac{\sum_{s_1,s_2,s_3,s_4,s_5 \in \{A,C,G,T\}} \mathbf{K}^5(s_3, s_1, s_2, s_3, s_4, s_5)}{\sum_{s,s_1,s_2,s_3,s_4,s_5 \in \{A,C,G,T\}} \mathbf{K}^5(s, s_1, s_2, s_3, s_4, s_5)}. \quad \text{(S3)}$$

Somatic hypermutagenesis of antibodies is the result of activation-induced deaminase (AID) enzyme activity that changes a random C:G base into a U:G base in B cell DNA. U:G mismatch can be repaired using UDG (uracil-DNA glycosylase) or MMR (DNA mismatch repair) machinery that forms diversity of hypermutations (Peled et al., 2008). Certain biological mechanisms of SHM occurrences were studied extensively. For example, Rogozin and Kolchanov (1992) observed specific hot/cold-spot DNA motifs for SHMs in immunoglobulin genes. Particularly, WRCY/RGYW where W = {A, T}, Y = {C, T}, R = {G, A} and later predicted more general WRCH/DGYW with H = {A, C, T} and D = {A, G, T} motifs are hot-spots for SHMs caused by weak hydrogen-bounds (Rogozin and Diaz, 2004). SYC/GRS (S = C, G) is a cold-spot motif caused by strong hydrogen-bounds (Bransteitter et al., 2004). The locality of AID enzyme activity has been emphasized. (Smith et al., 1996; Shapiro et al., 2003).

To simulate SHM, we modified a model proposed by Yaari et al. (2013). The model extends the notion of hot/cold-spots and suggests that a certain hierarchy of mutabilities exists following Smith et al. (1996) and Shapiro et al. (2003). The model is based on the mutability of a central base in each 5-mer of an antibody heavy chain and consists of two parts: a targeting model identifying if a mutation occurs in the variable part of an antibody and a substitution model providing an insight into what is this mutation. In order to avoid selection bias, the authors considered 5-mers where only synonymous substitutions of the central base are possible and inferred probabilities for other 5-mers. Unfortunately, synonymous substitutions constitute only a fraction of possible mutations. To overcome this issue, Yaari et al. (2013) proposed a special inference method to estimate parameters for the rest of 5-mers. Parameters for targeting and substitution models were inferred for 468 and 740 5-mers, respectively. However, the accuracy of this procedure was shown to be suboptimal (Yaari et al., 2013, Table 2). Additionally, some of the datasets that were used to estimate the parameters are derived from an error-prone 454 sequencing technology.

We re-estimated the parameters of this model and considered all 5-mers without limiting our scope to synonymous mutations. We also utilized three up-to-date repertoire sequencing datasets (all data were produced using the Illumina MiSeq platform):

- PRJNA349143. Time series of three individuals during influenza vaccination, both before and after vaccination.

- PRJNA395083. Bulk unsorted PBMC from peripheral blood of several healthy donors.
- A dataset of paired end sequences, added to increase power.

While the last dataset we used is not publicly available, we make the resulting k-mer model available publicly at `https://github.com/chaoszhang/immunosimulator/blob/master/kmerFreq.txt`.

From each dataset, we obtained a matrix of the size 1024×4, where each row corresponds to a distinct 5-mer and contains *# non-mutated occurrences* of this 5-mer and three possible *# nucleotide substitution occurrences*. To calculate this matrix for a given dataset, we found the closest V gene for every read and record the number of observed 5-mers in the gene and their corresponding mutated copies across the read. For any 5-mer K, the corresponding row of a constructed matrix can be viewed simultaneously as a value of *Binomial* and *Multinomial* distributions. *Binomial* distribution represents the number of occurred mutations among all occurrences of the 5-mer K, while *Multinomial* distribution indicates the number of mutations to specific bases among all occurred mutations. The parameters of these distributions indicate the mutability and substitution profiles for each 5-mer K. The 5-mer frequencies were combined across all these datasets to obtain the final matrix, available at `https://github.com/chaoszhang/immunosimulator/blob/master/kmerFreq.txt`.

2.3 Default parameters

Here we provide the actual default values used for several parameters that did not fit in Table 1 of the main paper.

2.3.1 BLOSUM.

The BLOSUM matrix table (Table S2) is obtained from `ftp://ftp.ncbi.nih.gov/blast/matrices/BLOSUM100`.

Table S2. BLOSUM table

	A	R	N	D	C	Q	E	G	H	I	L	K	M	F	P	S	T	W	Y	V
A	8	-3	-4	-5	-2	-2	-3	-1	-4	-4	-4	-2	-3	-5	-2	1	-1	-6	-5	-2
R	-3	10	-2	-5	-8	0	-2	-6	-1	-7	-6	3	-4	-6	-5	-3	-3	-7	-5	-6
N	-4	-2	11	1	-5	-1	-2	-2	0	-7	-7	-1	-5	-7	-5	0	-1	-8	-5	-7
D	-5	-5	1	10	-8	-2	2	-4	-3	-8	-8	-3	-8	-8	-5	-2	-4	-10	-7	-8
C	-2	-8	-5	-8	14	-7	-9	-7	-8	-3	-5	-8	-4	-4	-8	-3	-3	-7	-6	-3
Q	-2	0	-1	-2	-7	11	2	-5	1	-6	-5	2	-2	-6	-4	-2	-3	-5	-4	-5
E	-3	-2	-2	2	-9	2	10	-6	-2	-7	-7	0	-5	-8	-4	-2	-3	-8	-7	-5
G	-1	-6	-2	-4	-7	-5	-6	9	-6	-9	-8	-5	-7	-8	-6	-2	-5	-7	-8	-8
H	-4	-1	0	-3	-8	1	-2	-6	13	-7	-6	-3	-5	-4	-5	-3	-4	-5	1	-7
I	-4	-7	-7	-8	-3	-6	-7	-9	-7	8	2	-6	1	-2	-7	-5	-3	-6	-4	4
L	-4	-6	-7	-8	-5	-5	-7	-8	-6	2	8	-6	3	0	-7	-6	-4	-5	-4	0
K	-2	3	-1	-3	-8	2	0	-5	-3	-6	-6	10	-4	-6	-3	-2	-3	-8	-5	-5
M	-3	-4	-5	-8	-4	-2	-5	-7	-5	1	3	-4	12	-1	-5	-4	-2	-4	-5	0
F	-5	-6	-7	-8	-4	-6	-8	-8	-4	-2	0	-6	-1	11	-7	-5	-5	0	4	-3
P	-2	-5	-5	-5	-8	-4	-4	-6	-5	-7	-7	-3	-5	-7	12	-3	-4	-8	-7	-6
S	1	-3	0	-2	-3	-2	-2	-2	-3	-5	-6	-2	-4	-5	-3	9	2	-7	-5	-4
T	-1	-3	-1	-4	-3	-3	-3	-5	-4	-3	-4	-3	-2	-5	-4	2	9	-7	-5	-1
W	-6	-7	-8	-10	-7	-5	-8	-7	-5	-6	-5	-8	-4	0	-8	-7	-7	17	2	-5
Y	-5	-5	-5	-7	-6	-4	-7	-8	1	-4	-4	-5	-5	4	-7	-5	-5	2	12	-5
V	-2	-6	-7	-8	-3	-5	-5	-8	-7	4	0	-5	0	-3	-6	-4	-1	-5	-5	8

2.3.2 Starting and target sequences.

2.3.2.1 SARS-CoV2

The starting sequence $\hat{\Psi}$ is set to:

CAAATGCAGCTGGTGCAGTCTGGGCCTGAGGTGAAGAAGCCTGGGACCTCAGTGAAGGTCTCCT
GCAAGGCTTCTGGATTCACCTTTACTAGCTCTGCTGTGCAGTGGGTGCGACAGGCTCGTGGACAA
CGCCTTGAGTGGATAGGATGGATCGTCGTTGGCAGTGGTAACACAAACTACGCACAGAAGTTCCA
GGAAAGAGTCACCATTACCAGGGACATGTCCACAAGCACAGCCTACATGGAGCTGAGCAGCCTGA
GATCCGAGGACACGGCCGTGTATTACTGTGCGGCACCGCACTGCAGCGGCGGCAGCTGCCTCGAT
GCTTTTGATATCTGGGGCCAAGGGACAATGGTCACCGTCTCTTCA

and thus ζ_0 is

QVQLVQSGPEVKKPGTSVKVSCKASGFTFTSSAVQWVRQARGQRLEWIGWIVVGSGNTNYAQKF
QERVTITRDMSTSTAYMELSSLRSEDTAVYYCAAPHCSGGSCLDAFDIWGQGTMVTVSS.

In each replicate simulation $\boldsymbol{\zeta}_i$ and t_i are randomly chosen from Table S3.

Table S3. Names of antibodies in CoV-AbDab, heavy chain sequences (targets), and starting days of infection

**Name**	**Target Sequence**	**Day**
C005	QVQLVQSGPEVKKPGTSVKVSCKASGFTFTSSAVQWVRQAR<br>GQRLEWIGWIVVGSGNTNYAQKFQERVTITRDMSTSTAYME<br>LSSLRSEDTAVYYCAAPHCSGGSCLDAFDIWGQGTMVTVSS	0
COV2-2072	QMQLVQSGPEVKKPGTSVKVSCKTSGFTFTSSAIQWVRQAR<br>GQRLEWIGWIVVGSGNTNYAQKFQERVTITRDMSTSTAYME<br>LSSLRSEDTAVYYCAAPHCNRTSCYDAFDLWGQGTMVTVSS	41
Ab_58G6	QMQLVQSGPEVKKPGTSVKVSCKASGFTFSSSAVQWVRQAR<br>GQHLEWIGWIVVGSGNTNYAQKFQERVTLTRDMSTRTAYME<br>LSSLRSEDTAVYYCAAPNCNSTTCHDGFDIWGQGTVVTVSS	98
S2-E12	QVQLVQSGPEVKKPGTSVRVSCKASGFTFTSSAVQWVRQAR<br>GQRLEWVGWIVVGSGNTNYAQKFHERVTITRDMSTSTAYME<br>LSSLRSEDTAVYYCASPYCSGGSCSDGFDIWGQGTMVTVSS	163
B1-182-1	QMQLVQSGPEVKKPGTSVKVSCKASGFTFTSSAVQWVRQAR<br>GQRLEWIGWIVVGSGNTNYAQKFQERVTITRDMSTSTAYME<br>LSSLRSEDTAVYYCAAPYCSGGSCFDGFDIWGQGTMVTVSS	272
COVOX-253H55L	QVQLVQSGPEVKKPGTSVKVSCKASGFTFTTSAVQWVRQAR<br>GQRLEWIGWIVVGSGNTNYAQKFQERVTITRDMSTTTAYME<br>LSSLRSEDTAVYFCAAPHCNSTSCYDAFDIWGQGTMVTVSS	283
C597	QVQLVQSGPEVKKPGTSVKVSCKASGFTFTNSAVQWVRQSR<br>RQRLEWIGWIVVGSGNTNYAQKFQERVTITRDMSTSTAYME<br>LSSLRSEDTAVYYCAAVDCNSTSCYDAFDIWGQGTMVTVSS	430
AZD-8895	QMQLVQSGPEVKKPGTSVKVSCKASGFTFMSSAVQWVRQAR<br>GQRLEWIGWIVIGSGNTNYAQKFQERVTITRDMSTSTAYME<br>LSSLRSEDTAVYYCAAPYCSSISCNDGFDIWGQGTMVTVSS	458
CS102	QVQLVQSGPEVKKPGTSVKVSCKASGFTFPSSAVQWVRQAR<br>GQRLEWIGWIVVGSGNTNYAQKFQERVTITRDMSTSTAYME<br>LSSLRSEDTAVYYCAAPHCGGGSCYDGFDIWGQGTMVTVSS	504
Beta-47	QVQLVESGPEMKKPGTSVKVSCKASGFTFITSAVQWVRQAR<br>GQRLEWMGWIAVGSGNTNYAQKFQDRVTINRDMSTSTAYME<br>LSSLRSEDTAVYYCAAPHCNRTSCHDGFDIWGQGTMVTVSS	575
CZ-D7	QMQLVQSGPEVKKPGTSVKVSCKASGFTFTNSAMQWVRQAR<br>GQRLEWVGWIVVASGNANSARRFHDRVTITSDMSTSTAYLE<br>LSSLRSEDTAVYYCALNHCSNTTCLDGFDIWGQGTMVSVSS	609
R259-1B9	QMHLVQSGPEVKKPGTSVKVSCKASGFTFSSSAVQWVRQAR<br>GQHLEWIGWIVVGSGNTNYGQKFQERVTITRDLSTSTVYME<br>LISLRSEDTAVYFCAAPYCTGGSCFDAFDIWGQGTMVTVSS	641
Omi-12	EVQLVESGPEVKKPGTSVKVSCKASGFSFSMSAMQWVRRAR<br>GQRLEWIGWIVPGSGNANYAQKFQERVTITRDESTNTGYME<br>LSSLRSEDTAVYYCAAPHCNKTNCYDAFDIWGQGTMVTVSS	743
BD57-049	QMQLVQSGPEVKKPGTSAKVACQASGFTFYSSAIQWVRQAR<br>GQRLEWIGWIVVGSGNTNYAEEFQERVTITRDMSTSTAYME<br>LSSLRSGDTAVYYCAAPHCNRTSCYDGFDIWGQGTMVTVSS	785

2.3.2.2 Influenza

The starting sequence $\hat{\Psi}$ is set to:

CAGGTGCAGCTGCAGGAGTCGGGCCCAGGACTGGTGAAGCCTTCACAGACCCTGTCCCTCACCT
GCACTGTCTCTGGTGGCTCCATCAGCAGTGGTGGTTACTACTGGAGCTGGATCCGCCAGCACCCA
GGGAAGGGCCTGGAGTGGATTGGGTACATCTATTACAGTGGGAGCACCTACTACAACCCGTCCCT
CAAGAGTCGAGTTACCATATCAGTAGACACGTCTAAGAACCAGTTCTCCCTGAAGCTGAGCTCTG
TGACTGCCGCGGACACGGCCGTGTATTACTGTGCGAGAGCGCGCGTCAATAGGGATATTGCGTAC
GGCAACTGGTTCGACCCCTGGGGCCAGGGGACCCTGGTCACCGTCTCCTCA

and thus ζ_0 is

QVQLQESGPGLVKPSQTLSLTCTVSGGSISSGGYYWSWIRQHPGKGLEWIGYIYYSGSTYYNPS
LKSRVTISVDTSKNQFSLKLSSVTAADTAVYYCARARVNRDIAYGNWFDPWGQGTLVTVSS.

$\boldsymbol{\eta}_i$, $\boldsymbol{\zeta}_i$, and t_i are given in Table S4.

Table S4. Flu accession numbers, CDRs of target sequences, and starting days of infection

i	Accession	Target CDR1	Target CDR2	Target CDR3	Day
1	AAK70482.1	SGGYY	IGYIYYSGSTYYNPSL	ARARVNRDIAYGNWFDP	0
2	AAK70478.1	CWWVP	WWCHCGWCNVXXNIXF	ARARVNREXAYGNWFZA	182
3	ABL76892.1	WWWXX	XGYVYYSGSDYYDPSL	VKVKVNKEVVYGNWFEA	365
4	AFP83103.2	WWWAB	TBYVYYSGSDYYDXSL	VKVKINKEVVYGNWFEA	398
5	AFP83094.2	WWWGX	TGYVYYSGSDYYDXSL	VKVKVNKEVVYGNWFEQ	431
6	AFP83095.2	WWCPP	WWCHCAWXBTXXBISL	ARARVNRELAYGNWFEA	464
7	AFP83197.2	WWCPP	WWCHCZWYZVXXBISF	ARARVNRELAYGNXFEA	497
8	AFP83098.2	WWWAX	AGYVYYSGTDYYDBSL	VKVKINKEVVYGBWFEZ	530
9	AFP83100.2	WWWPK	SXHVYYSGSDYYDXSL	VKVKVNKEVVYGNWFEA	564
10	AAO38870.2	WWCPP	WWCHCCWXBVXYBXSY	ARARVNRELAYGNWFZA	597
11	AFP83199.2	WWLPP	WWCHCEWLHVXXXIXY	ARARVNRELAYGNWFZA	630
12	ABL76881.1	WLWCG	KXYVYYSGSQFYDASL	VKVKLNKEVVYGNWFZL	663
13	AFP83097.2	WCWCG	CRWVYYXXSDYYDIXL	VKVKINKEVVYGDWFEQ	696
14	AFP83202.2	WXYXY	TGYVYYSGSDYYDPSL	VKVKMNKEVVYGNWFEA	730
15	AFP83201.2	WWVPP	WWCNCCWFBTXXXLSF	ARARVNRELAYGNWFEA	763
16	AFP83118.2	WYYXD	TGYVYYSGSDYYBPSL	VKVKLNKEVVYGNWFZK	796
17	AFP83200.2	WWCPP	WWCHCCYIBVXXBXSY	ARARVNRELAYGNWFZA	829
18	AFP83107.2	WWCPP	WWCHCCYVBTXXBXSF	ARARVNRELAYGNWYZA	862
19	AFP83112.2	WFWDG	XKWVYYSGSDYYDXSL	VKVKINKZVVYGNWFEQ	895
20	AFP83115.2	WWCPP	WWCHCCQIBTXXBXSF	ARARVNRELAYGNWFZG	929
21	AFP83114.2	WPWGD	XGYVHYSRSDYYDPSL	VKVKXNKZVVYRNWFEP	962
22	AFP83110.2	WWCPD	WWCHCCWIDWXXBXXY	ARARVNRZLAYRNWFEA	995
23	AFP83105.2	WYWGN	GCXLYYSGSDYYDPSL	IKVKIDKELVYGDWFZV	1028
24	AFP83106.2	WWCPP	WWCHCCWVWWNEGLXB	GXXRXXRDLAYGNWYXA	1061
25	AFP83127.2	WFWBG	TGYLYYSGSDYYDASL	IKVKXNKELVYGNWFET	1095
26	AFP83124.2	WCWCG	BGYLYYSGSDYYBFSL	IKVCIBKEMVYGBWFET	1216
27	AFP83130.2	WWHPP	WWCHCCWRBCXXXXSF	ARARVNRSLAYGNWFEA	1338
28	AFP83134.2	WBYXY	TGYVYYSGSDYYBPSL	VKVKMNKEVVYGNWFEA	1460
29	AFP83131.2	WWHPP	WWCHCCWRBLXXXXSF	ARARVNRZLAYGNWFEA	1581
30	AFP83135.2	PPYGD	PGKVYYSRSDYYDDSL	IKVKXNKYVVYRNWFEK	1703
31	AFP83150.2	HPYGD	PGBVYYSRSDYYDBSL	VKVKINKZVVYRNWFEK	1825
32	AFP83206.2	HPYGD	PPHCYYSRSDYYDBSL	VKVKXNKFVVYRNWFEZ	1946
33	AFP83147.2	HPYGD	PGHVYYSRSDYYDPSL	IKVKINBXVVYRNWFEK	2068
34	AFP83154.2	WXXAY	PGYVYYSGSDYYDPSL	VKVKMNKEVVYGNWFEP	2190
35	AFP83155.2	LPYGD	PGHVYYSRSDYYDDSL	VKVKLBKIVVYRNWFEK	2281
36	AFP83160.2	HPYGD	PGHVYYSRSDYFDDSL	VKVKXNKZVVYRNWFEK	2372
37	AFP83159.2	HPYGD	PGHVYYSHSDYYDDSL	IKVKXNKZVVYRNWFEK	2463
38	AFP83166.2	WEHGY	XGYVYYSGSDYYDPSC	VKVKMNKEVVYGNWFEP	2555
39	AFP83173.2	WBIMY	LGFVYYSGSDYYBPSL	VKVKMNKZVVYGNWFZA	2920
40	AFP83163.2	WPIFY	LGYVYYSGSBYYBPSL	VKVKMNKZIVYGNWFZA	3011
41	AFP83170.2	YZIMY	LGYVYYSASDYYBPSL	VKVKMNKEIVYGNWFEA	3102
42	AFP83174.2	YPIMY	SGYVYYSGSDYYBPSL	VKVKMNKEVVYGBWFEA	3193
43	AFP83184.2	ZSZYY	TDYVYYSGIDYYTPSL	VKVKMNKEVVYDYWFEP	3285
44	AFP83185.2	BBGYY	TDYVYYSGIDYYYPSL	VKVKMTKEVVYDYWFZP	3345
45	AFP83181.2	EBAYY	TDYVYYSGVDYYEPSL	VKVKMNKEVVYDYWFEP	3406
46	AFP83208.2	WDIPY	LGYVYYSASDYYBPSL	VKVKMNKZVVYGNWFZA	3467
47	AFP83178.2	FKIMY	LGYVYYSGSDYYDPSL	VKWKMBKZVYYGNWFZA	3528
48	AFP83177.2	YEIMW	LGFVYYSGSDYYBPSL	VKVKMNKZAVYGNWFZA	3589
49	AJK04689.1	DDGYY	TDYVYYSGIDYYEPSL	VKMKMAKZTVYDYWFZP	3650
50	AJK04818.1	EBFYY	TDYVYYSGVDYYCPSI	VKVKMBKEVVYDYWLEP	3832
51	AJK04119.1	ZDPYY	TDYVYYSGIDYYBPSL	VKVKMRKEVVYDHWFEP	4015
52	AFP83190.2	DDDYF	TDYVYYSGIDYYWPSL	VKVKMTKZVVYDYWFZP	4075
53	AJK05467.1	DDRYY	TDYIYYSGIDYYKPSL	VKVKMSKZVVYDYWFZP	4136
54	AJK05084.1	DDGYY	TDYIFYSGITYYVPXL	VKVKMSKEVIYDHWFZP	4197
55	AJK04964.1	DDGYY	CDYXFYSGIDYYSPSC	VKVKMSKEVVYDYWFEP	4258
56	AJK05278.1	EDFYY	TDYVWYTGIDYYXPXL	VKVKMVKXVVXDYWFZP	4319

2.4 Evaluation metrics

2.4.1 Notations.

For a rooted tree T, we let $\mathbf{L}_T$ be the set of leaves and $\mathbf{I}_T$ be the set of internal nodes. For each node v of T, let $\mathcal{C}(v)$ be the set of its children. We define $\phi(v)$ as the set of node labels of labeled nodes below v. Also, for any *set* of nodes V, we define $\phi(V) = \{\phi(v) : \phi(v) \neq \emptyset, v \in V\}$ and $\phi(T) = \phi(\mathbf{I}_T \cup \mathbf{L}_T)$. For a set of nodes V and a set of labels $\mathbf{\Phi}$, $\phi(V) \restriction \mathbf{\Phi} = \{\mathbf{\Phi'} \cap \mathbf{\Phi} : \mathbf{\Phi'} \cap \mathbf{\Phi} \neq \emptyset, \mathbf{\Phi'} \in \phi(V)\}$. For labeled nodes Ψ_i and Ψ_j, let $\boldsymbol{U}_T(i,j)$ be the number of edges between the node Ψ_i in T and the MRCA of Ψ_i and Ψ_j in T.

2.4.2 Characterizing a clonal tree

We define a set of metrics for characterizing properties of simulated trees in terms of their topology, branch length, and distribution of labeled nodes (Table S5). Some of these metrics are motivated by similar ones on phylogenetic trees, but are adjusted to allow sampled internal nodes and multifurcations. For example, to measure tree balance, we extend the definition of the number of cherries but allow modifications (our definition reduces to the traditional definition when the tree is binary). Other metrics (e.g., percent internal samples) are only meaningful for clonal trees and are meant to quantify the deviation of a clonal tree from phylogenetic trees.

2.4.3 Comparing trees

Many metrics exist for comparing phylogenetic trees. However, in the presence of polytomies and sampled ancestral nodes, the classic metrics need to be amended. Here, we generalize several existing metrics and introduce new ones. All metrics are defined over a simulated tree R and a reconstructed tree E, both induced down to include all labeled nodes (i.e., removing unlabeled nodes if less than two of their children have any labeled descendants). See Table S6 for precise definitions of metrics.

2.4.3.1 RF-related.

We refer to the set of labeled nodes under some subtree as a cluster. We define False Discovery Rate (FDR) as the percentage of clusters in E that are not in R, False Negative Rate (FNR) as the percentage of clusters in R that are not in E, and Robinson-Foulds cluster distance (RF) as the number of clusters in either but not both trees. Note that unlike traditional Robinson and Foulds

Table S5. Properties of a clonal tree T.

Property	Definition
Internal sample (%)	The percentage of labeled nodes in set $\mathbf{I}_T$.
Bifurcation index	Defined as $\frac{\lvert\mathbf{I}_T\rvert}{\lvert\mathbf{L}_T\rvert-1}$ equals 1 for bifurcating trees and ≈ 0 for the star tree.
Sample depth	The average depth of labeled nodes in T.
Balance (cherry)	Half the sum over all leaves of the fraction of their siblings that are leaves. $\sum_{v\in\mathbf{I}_T} \binom{\lvert\mathcal{C}(v)\cap\mathbf{L}_T\rvert}{2}/(\lvert\mathcal{C}(v)\rvert-1)$ where $0/0 \doteq 1/2$
Single mutation branches (%)	The percentage of branches with length one.
Accumulated mutations (avg)	The average depth (path length to the root) of all labeled nodes of tree T.
Accumulated mutations (sum)	The summation of branch lengths of all branches of tree T.
Mutations per branch	The average branch length of tree T.

The last four metrics require branch length (in mutation unit) on the tree.

(1981) distance, here, internal nodes can also have labels, and we define the metric based on clusters in a rooted tree instead of bipartitions in an unrooted tree. Moreover, the singleton clusters are trivial when all labeled nodes are leaves; however, when there are labeled internal nodes, including or excluding singletons can make a difference. Thus, we also define FPR FNR, and RF distance when excluding singleton clusters.

2.4.3.2 Triplet-based.

We define *triplet discordance* (TD) as the number of trees induced by triples of *labeled* nodes (leaf or internal) where the topology in the simulated tree and the reconstructed tree differ. We define the *triplet edit distance* (TED) as the summation over all triplets of the labeled nodes of cluster RF distance between the two trees induced to the triplet. Intuitively, it is the sum of the minimum number of branch contractions and resolutions required to covert a triplet in R to a triplet in E, summed over all triplet.

2.4.3.3 Path discordance.

Patristic discordance for a pair of labeled nodes Ψ_i and Ψ_j is defined as the difference between the number of branches in the path between Ψ_i and Ψ_j on two trees R and E. The patristic discordance (PD) between R and E is the summation of the Patristic discordance over all pairs of labeled nodes (internal or leaf). We define the MRCA discordance for an ordered pair of labeled nodes Ψ_i and Ψ_j as the difference between the number of branches in the path between Ψ_i and its MRCA with Ψ_j when computed from trees R and E. The MRCA discordance (MD) between the two trees is the summation of MRCA discordance over all ordered pairs of labeled nodes.

The FNR and FDR metrics are already normalized. To normalize other metrics, for each experimental condition, we create a control tree by randomly permuting labels of the true tree. We then normalize scores (other than FNR and FDR) of a reconstruction method by dividing it by the average score of replicates of the control method.

Computing FNR, FDR, and RF metrics takes $O(\varsigma)$ time with hashing and randomization (algorithm S4). Triplet-based metric can be easily computed in $O(\varsigma^3)$ time with simple preprocessing and iterating over all triplets. Both PD and MD take $O(\varsigma^2)$ time with preprocessing that computes distances to MRCAs.

Table S6. Metrics for comparing the reference simulated tree R to estimated tree E.

Metric	AB	Definition
False discovery rate	FDR	$\lvert\phi(E) \setminus \phi(R)\rvert / \lvert\phi(E)\rvert$
FDR no singletons	FDR*	$\lvert\phi(\mathbf{I}_E) \setminus \phi(\mathbf{I}_R)\rvert / \lvert\phi(\mathbf{I}_E)\rvert$
False negative rate	FNR	$\lvert\phi(R) \setminus \phi(E)\rvert / \lvert\phi(R)\rvert$
FNR no singletons	FNR*	$\lvert\phi(\mathbf{I}_R) \setminus \phi(\mathbf{I}_E)\rvert / \lvert\phi(\mathbf{I}_R)\rvert$
RF cluster distance	RF	$\lvert\phi(R) \cup \phi(E)\rvert - \lvert\phi(R) \cap \phi(E)\rvert$
RF cluster distance no singletons	RF*	$\lvert\phi(\mathbf{I}_R) \cup \phi(\mathbf{I}_E)\rvert - \lvert\phi(\mathbf{I}_R) \cap \phi(\mathbf{I}_E)\rvert$
Triplet discordance	TD	$\lvert\{\mathbf{\Phi} : \phi(R) \upharpoonright \mathbf{\Phi} \neq \phi(E) \upharpoonright \mathbf{\Phi}, \mathbf{\Phi} \subset \{\Psi_1, \ldots, \Psi_\varsigma\}, \lvert\mathbf{\Phi}\rvert = 3\}\rvert$
Triplet edit distance	TED	$\sum_{\mathbf{\Phi} \subset \{\Psi_1,\ldots,\Psi_\varsigma\}, \lvert\mathbf{\Phi}\rvert=3} \lvert(\phi(R) \upharpoonright \mathbf{\Phi}) \cup (\phi(E) \upharpoonright \mathbf{\Phi})\rvert - \lvert(\phi(R) \upharpoonright \mathbf{\Phi}) \cap (\phi(E) \upharpoonright \mathbf{\Phi})\rvert$
MRCA discordance	MD	$\sum_{i,j \in [\varsigma]} \lvert\boldsymbol{U}_R(i,j) - \boldsymbol{U}_E(i,j)\rvert$
Patristic distance	PD	$1/2 \sum_{i,j \in [\varsigma]} \lvert\boldsymbol{U}_R(i,j) + \boldsymbol{U}_R(j,i) - \boldsymbol{U}_E(i,j) - \boldsymbol{U}_E(j,i)\rvert$

3 SUPPLEMENTARY FIGURES

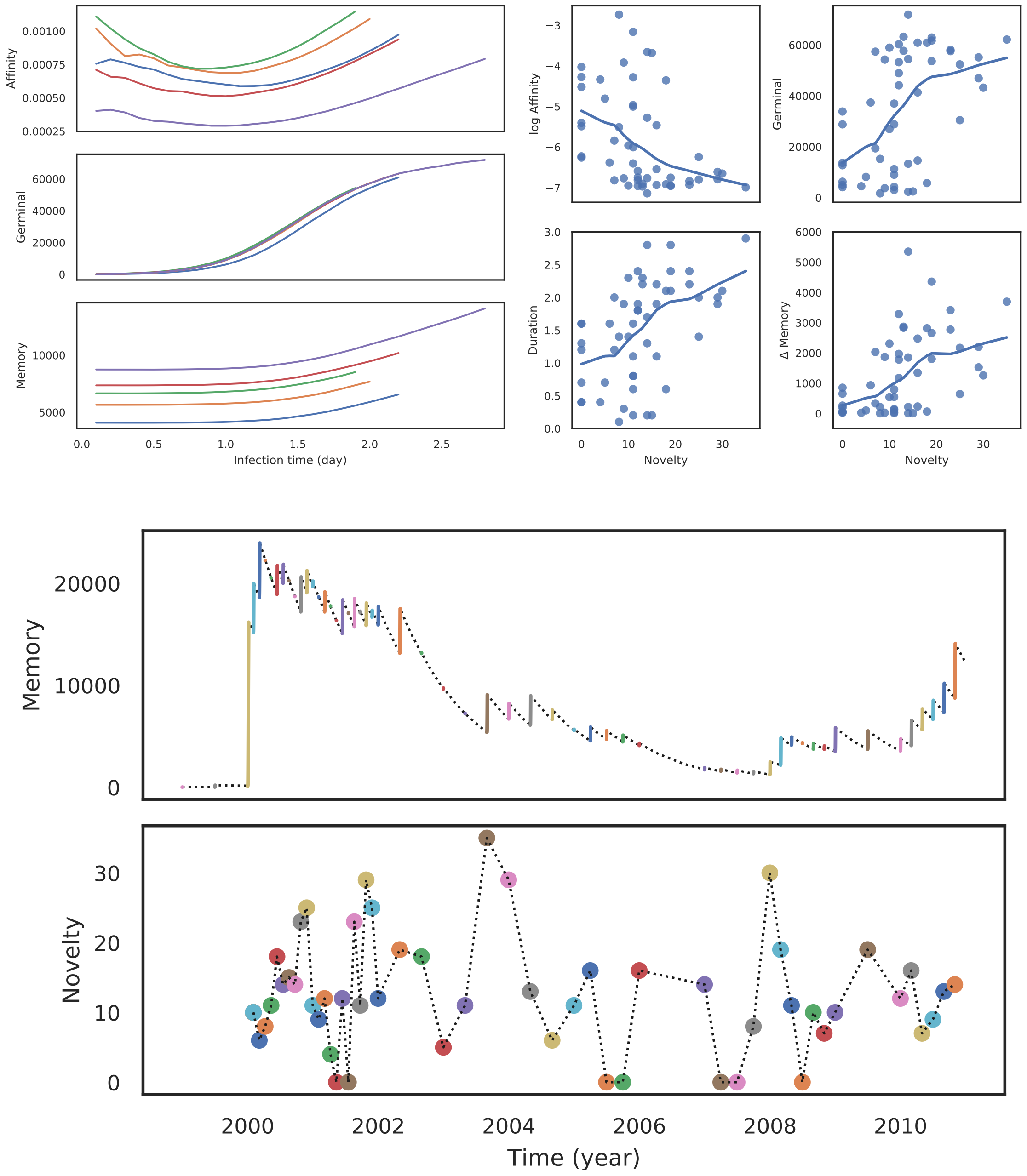

Figure S1. a) Log average affinity of activated cells to the current infection target at the end of the infection, the number of activated cells at the end of the infection, and the duration of infection by novelty of the target of one simulation under default conditions, showing the last five rounds as examples. b) Average affinity of activated cells to current infection target, the number of activated cells, and the number of memory cells by time after infection starts for the last five infections of one simulation under default conditions. Lines are fitted using the LOWESS (locally weighted scatter plot smoothing) algorithm. c) Number of memory cells and novelty of infections by time. Dormant stages are indicated by dotted lines.

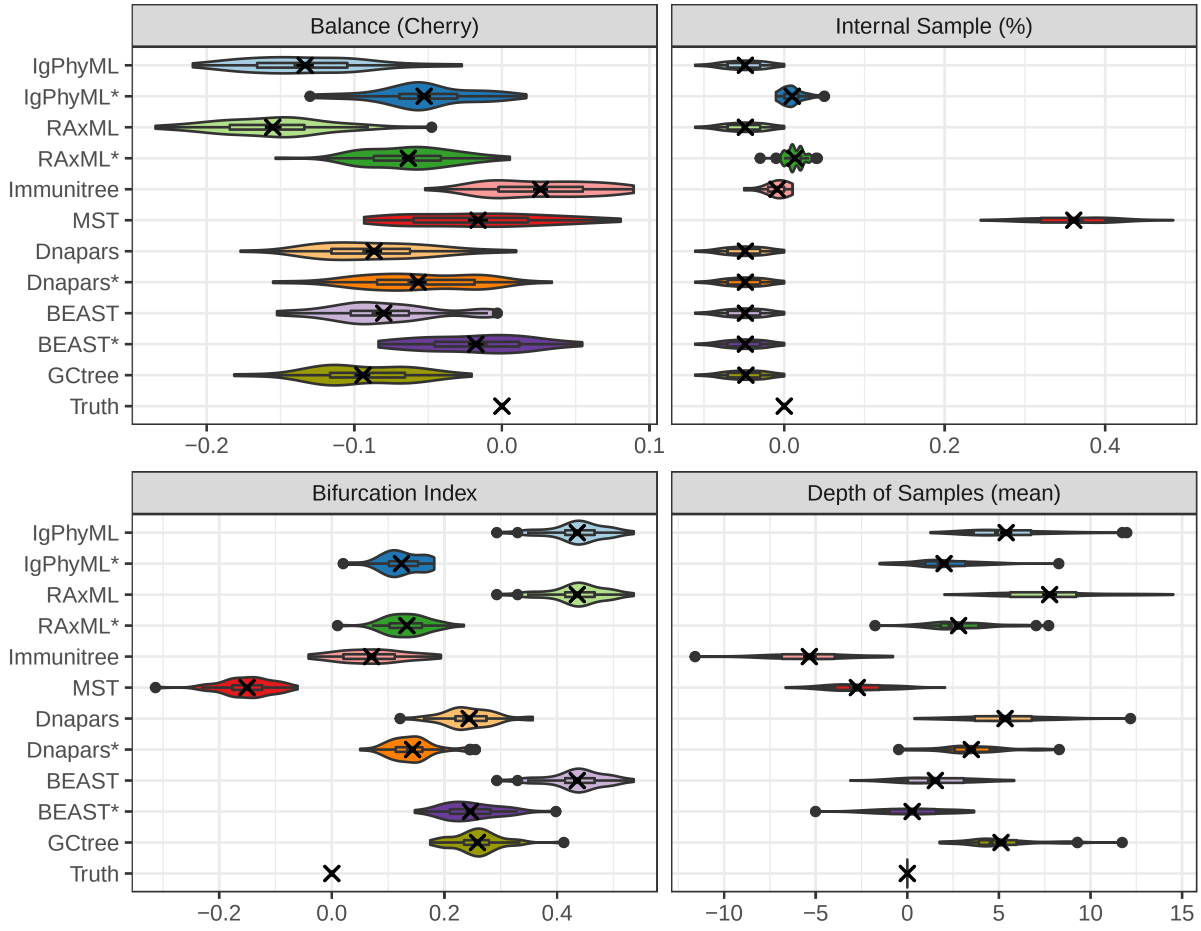

Figure S2. Property of the estimated tree in relative to the corresponding true tree (estimated minus tree) for default parameters of SARS-Cov2 dataset.

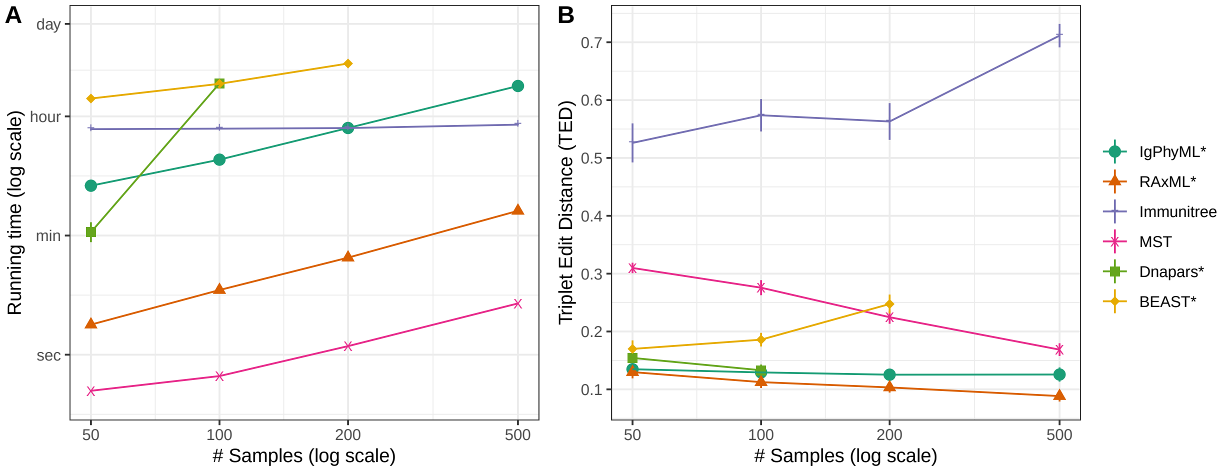

Figure S3. **(A)** Running time and **(B)** triplet edit distance (TED) of various reconstruction methods on SARS-CoV2 simulations under different sample sizes (50 replicates).

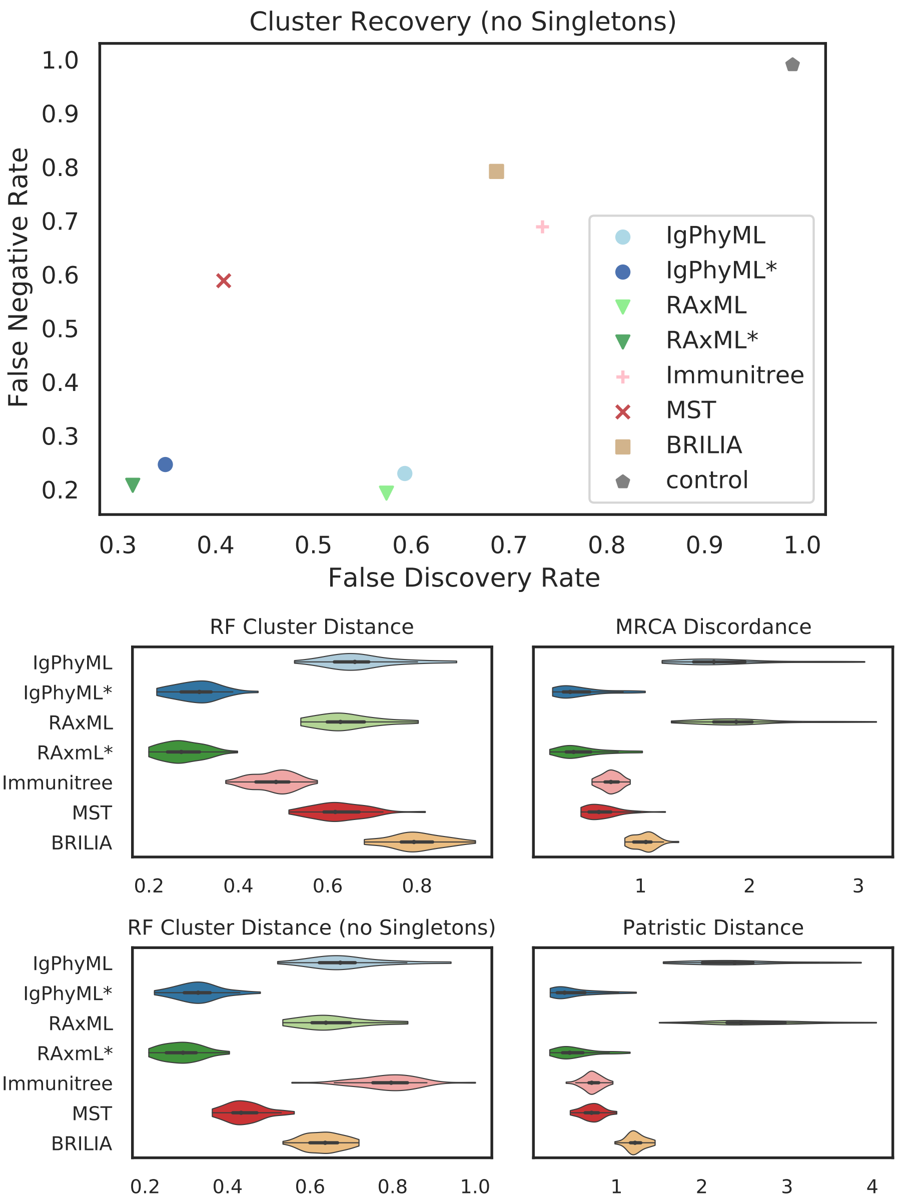

Figure S4. Top: FNR* and FPR* rates excluding singletons by reconstruction methods on simulations under default conditions; Bottom: Normalized Robinson-Foulds cluster distance with and without singletons (RF and RF *), MD and PD.

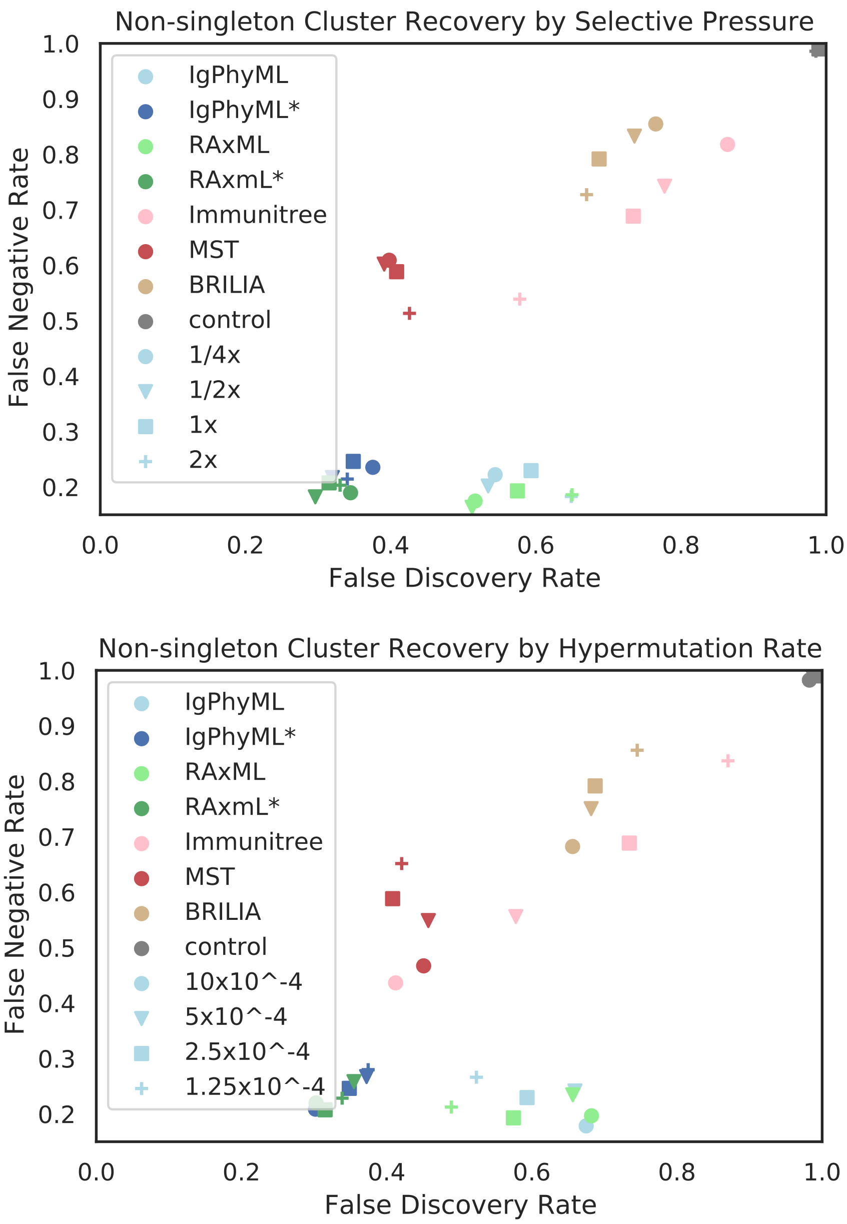

Figure S5. Impact of selective pressure A (a) and mutation rate μ (b) on tree inference error by FDR* and FNR*.

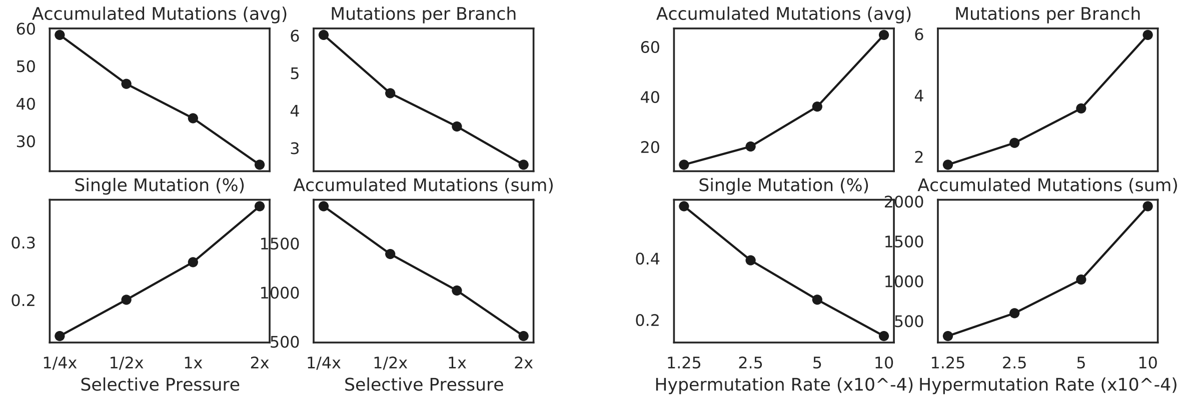

Figure S6. Impact of selective pressure A (left) and mutation rate μ (right) on sequence-based branch length properties on true trees. $\mu = 5 \times 10^{-4}$ in (a-d) and $A = 0.1$ in (e-h).

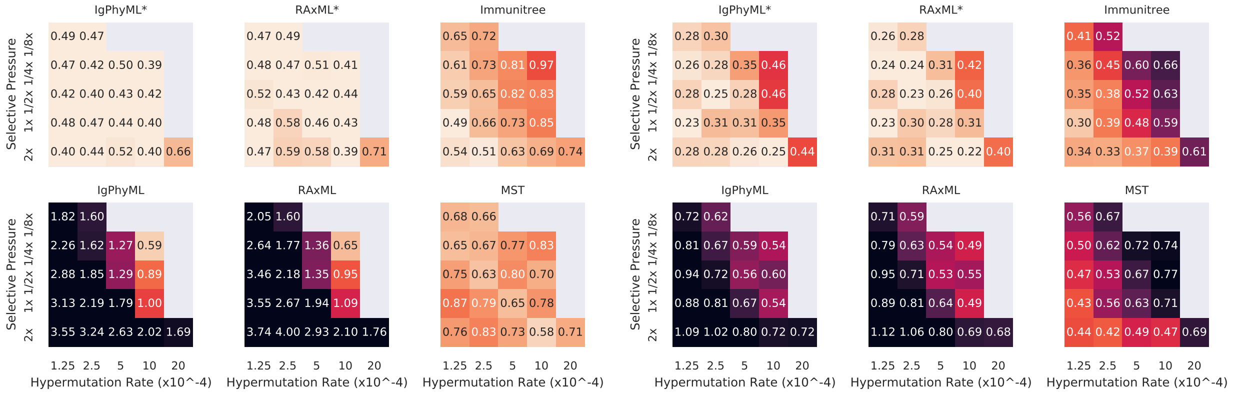

Figure S7. For varying levels of selective pressure (A), rate of hypermutation (μ), and reconstruction methods, we show MD error (left), and RF error (right). Under some conditions, reconstructed trees from phylogenetic methods are worse than random permuting labels of true tree because both MD and RF (to a lesser degree) severely penalizes resolution of multifurcated nodes.

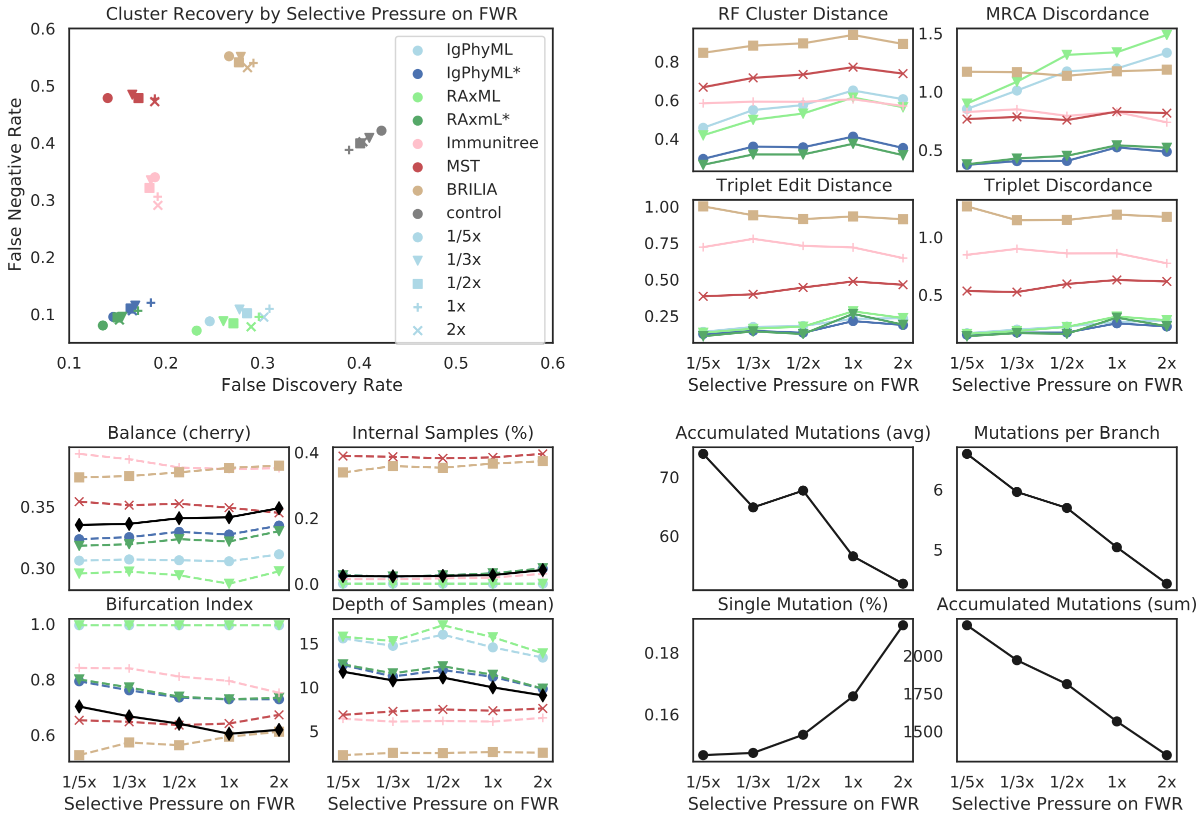

Figure S8. a) FNR versus FDR, b) Robinson-Foulds cluster distance (RF), MRCA Discordance (MD), triplet edit distance (TED), and triplet discordance (TD) by BLOSUM weight multiplier of framework region (w_f) and reconstruction methods. c) Properties of true (black) and reconstructed trees by BLOSUM weight multiplier of framework region (FR). d) Properties of true trees.

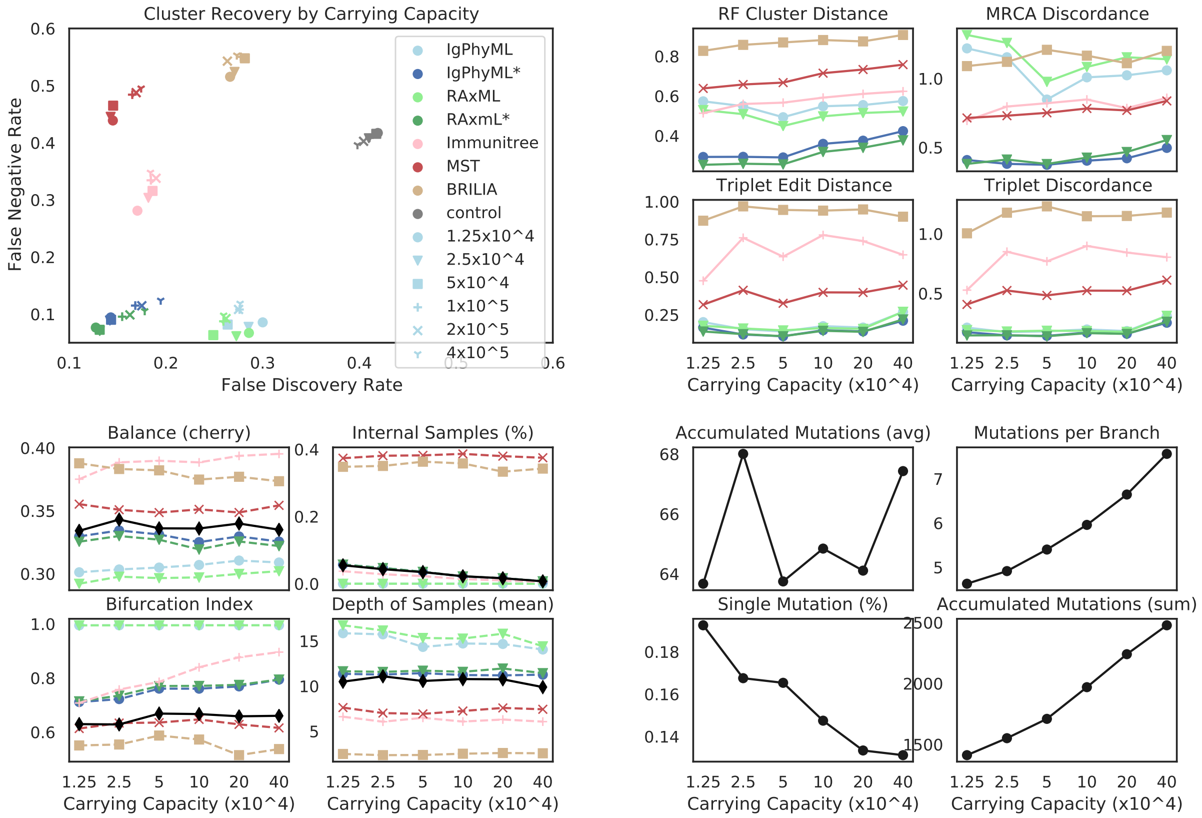

Figure S9. a) FNR versus FDR, b) Robinson-Foulds cluster distance (RF), MRCA Discordance (MD), triplet edit distance (TED), and triplet discordance (TD) by germinal center capacity (C) and reconstruction methods. c) Properties of true (black) and reconstructed trees by carrying capacity of germinal center of FR. d) Properties of true trees.

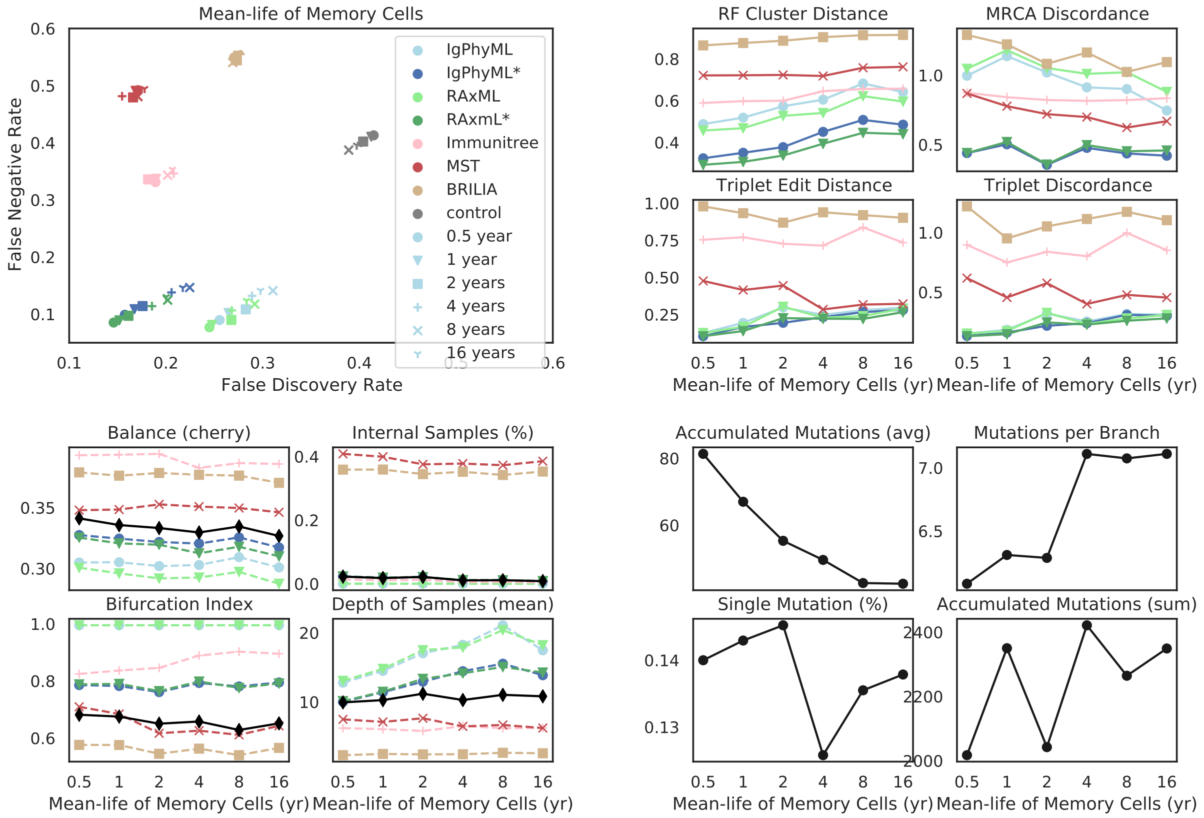

Figure S10. a) FNR versus FDR, b) Robinson-Foulds cluster distance (RF), MRCA Discordance (MD), triplet edit distance (TED), and triplet discordance (TD) by mean memory cell life-time ($^1/_{\lambda'_d}$) and reconstruction methods. c) Properties of true (black) and reconstructed trees by memory cell life (mean). d) Properties of true trees.

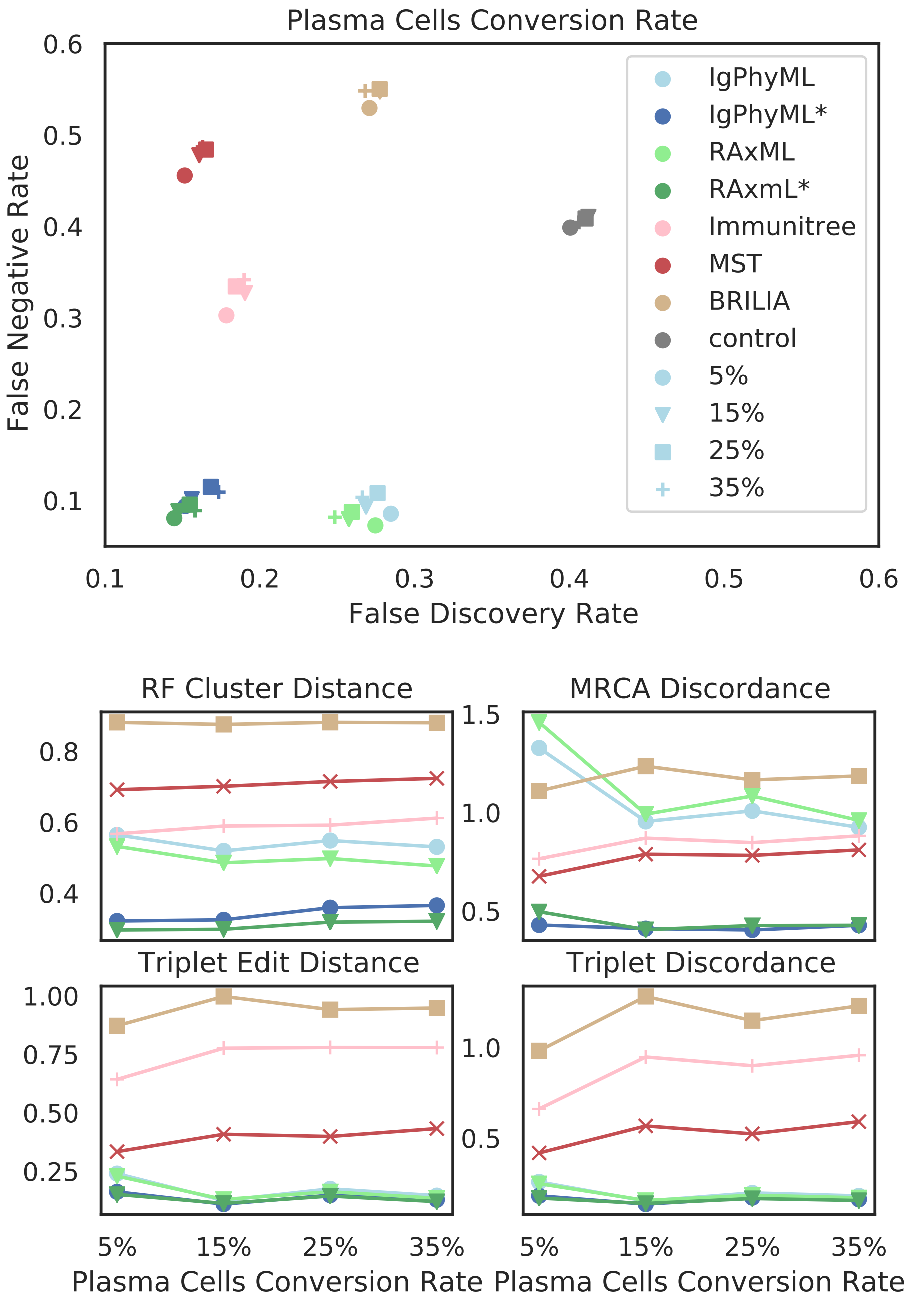

Figure S11. a) FNR versus FDR, b) Robinson-Foulds cluster distance (RF), MRCA Discordance (MD), triplet edit distance (TED), and triplet discordance (TD) by fraction of activated cells turning into plasma cell per cell division (ρ_p).

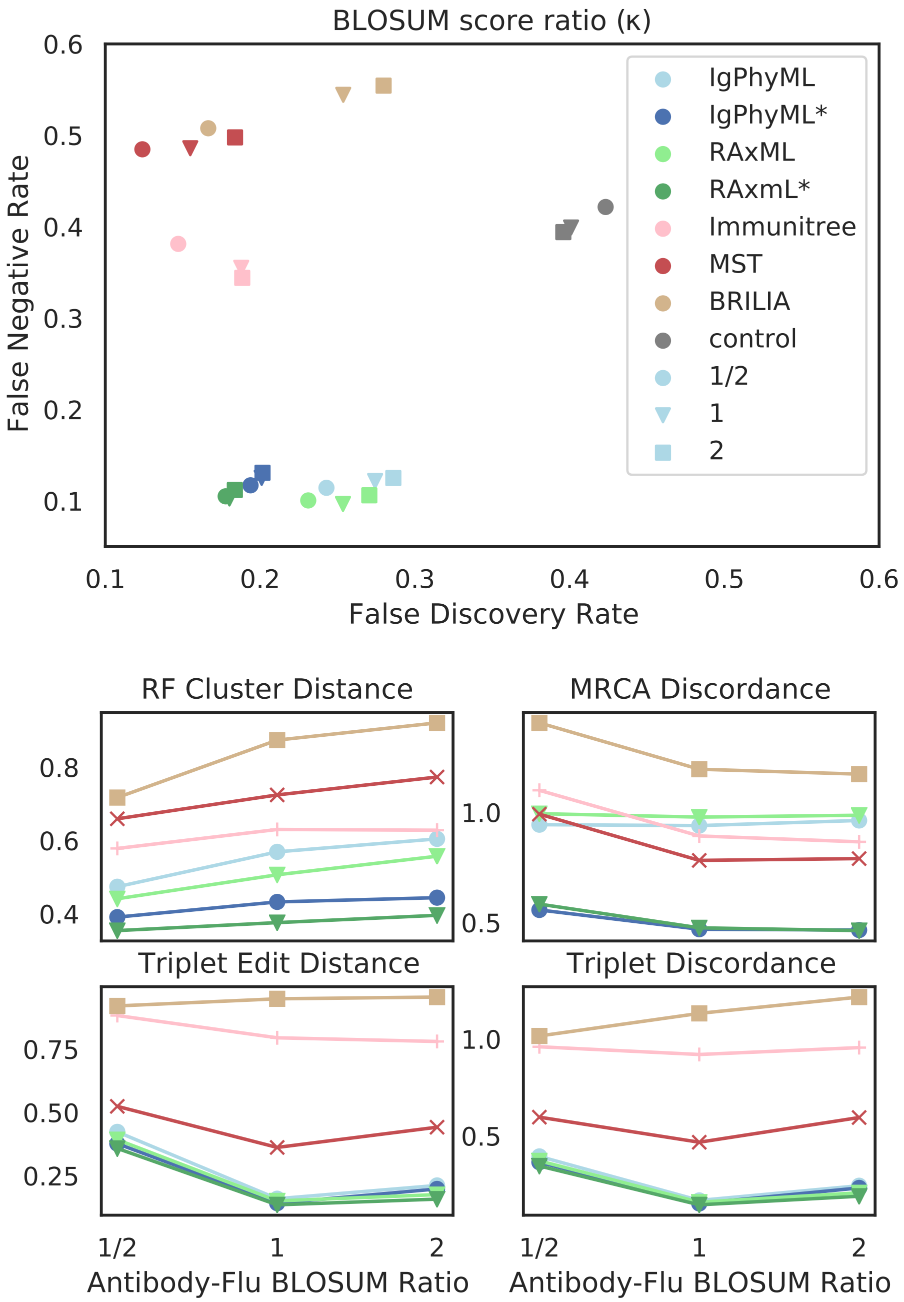

Figure S12. a) FNR versus FDR, b) Robinson-Foulds cluster distance (RF), MRCA Discordance (MD), triplet edit distance (TED), and triplet discordance (TD) by BLOSUM score ratio of antibody-coding sequences to antigen sequences (κ)

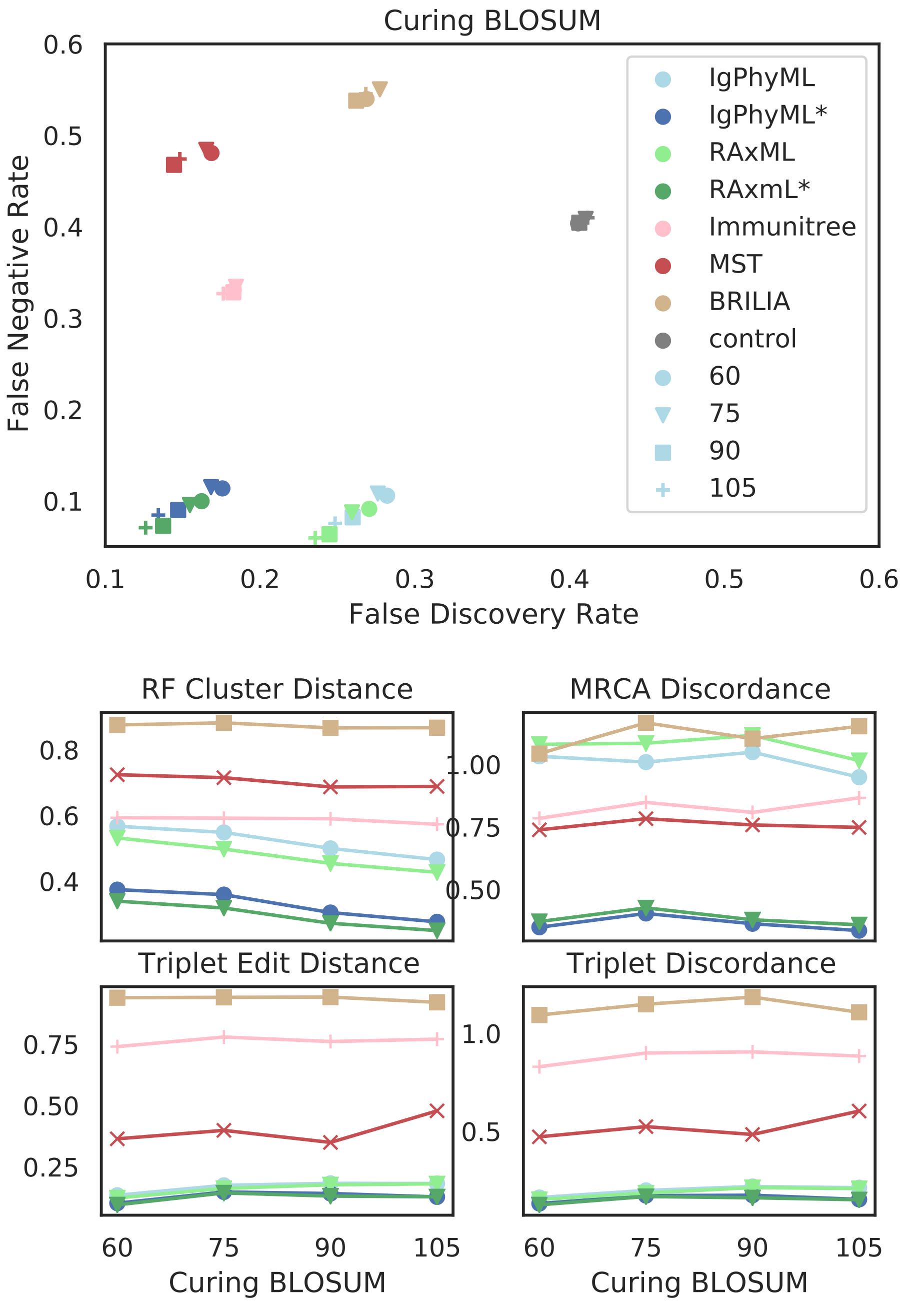

Figure S13. a) FNR versus FDR, b) Robinson-Foulds cluster distance (RF), MRCA Discordance (MD), triplet edit distance (TED), and triplet discordance (TD) by BLOSUM score of activated cell antibody-coding sequences that leads to cure (Δ_0').

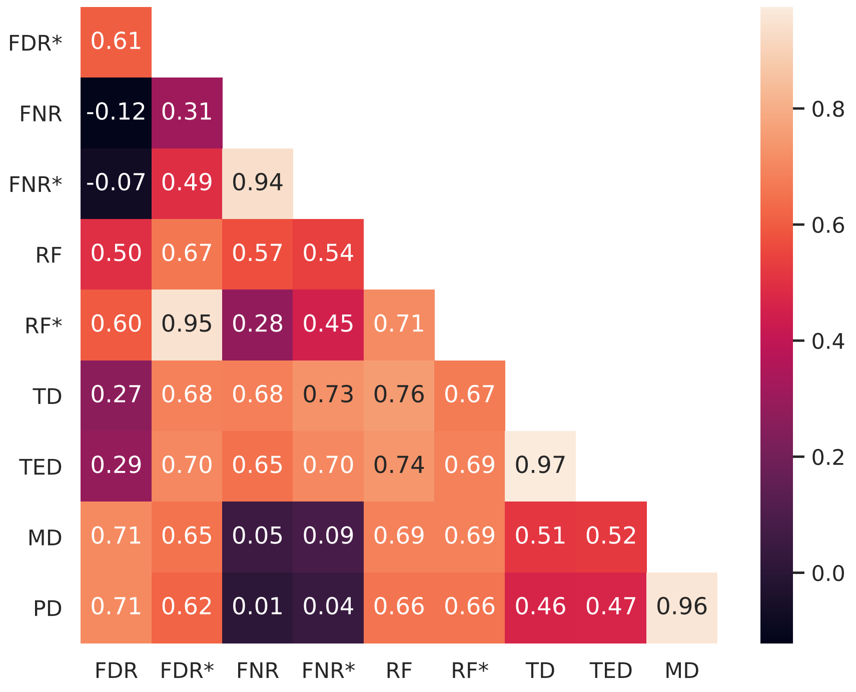

Figure S14. Correlations of evaluation metrics. For each replicate of each simulation condition, we compute Spearman's rank correlation coefficient of the reconstruction method for each pair of evaluation metrics. Here, we show the average coefficient over all replicates of all simulation conditions.

4 SUPPLEMENTARY ALGORITHMS

Algorithm S1 Simulating the next event and update time and S accordingly. Before running this procedure, we have computed $\mathbf{S}$ and $\theta_\alpha = \sum_{i \in S} \mathbf{x}_i^\alpha$ for all α from the previous calls to this function (i.e., previous time steps). For each α, we have also built an interval tree T_α on leafset S and each node v storing the summation of $\mathbf{x}_i^\alpha$ for each leaf i under v.

procedure SAMPLETREE(α, v)
  if v is a leaf node **then**
    return v
  else
    $L \leftarrow$ the sum of $\mathbf{x}_i^\alpha$ for each leaf i under left child of v
    $R \leftarrow$ the sum of $\mathbf{x}_i^\alpha$ for each leaf i under right child of v
    $O \leftarrow$ the outcome of a flip of a biased coin with probability of being head $\frac{L}{L+R}$
    if $O =$ Head **then**
      return SAMPLETREE(α, the left child of v)
    else
      return SAMPLETREE(α, the right child of v)
procedure SIMULATINGONEEVENT
  time $\leftarrow$ time + a random sample from exponential distribution where $\lambda = \frac{\sum_{\alpha,\beta \in \mathbf{\Gamma}} (P_{\alpha,\beta} \mathbf{S}^\beta \theta_\alpha)}{\sum_{\beta \in \mathbf{\Gamma}} Q_\beta \mathbf{S}^\beta}$
  $(\alpha, \beta) \leftarrow$ a random sample from distribution $Pr(\alpha, \beta) = \frac{P_{\alpha,\beta} \mathbf{S}^\beta \theta_\alpha}{\sum_{\bar{\alpha},\bar{\beta} \in \mathbf{\Gamma}} (P_{\bar{\alpha},\bar{\beta}} \mathbf{S}^{\bar{\beta}} \theta_{\bar{\alpha}})}$
  $i \leftarrow$ SAMPLETREE(α, the root of $T\alpha$)
  $E \leftarrow$ a sample from $Pr(E = \text{Birth}) = \frac{\mathcal{B}_{\alpha,\beta}}{P_{\alpha,\beta}}$, $Pr(E = \text{Death}) = \frac{\mathcal{D}_{\alpha,\beta}}{P_{\alpha,\beta}}$, $Pr(E = \text{Transformation}) = \frac{\mathcal{T}_{\alpha,\beta}}{P_{\alpha,\beta}}$
  if $E =$ Birth **then**
    $(j, k) \leftarrow$ a sample from distribution of outcomes of birth event of i
    $\mathbf{S} \leftarrow \mathbf{S} + \mathbf{x}_j + \mathbf{x}_k$
    $S \leftarrow S \cup \{j, k\}$
    for $\alpha \in \mathbf{\Gamma}$ **do**
      $\theta_\alpha \leftarrow \theta_\alpha + \mathbf{x}_j^\alpha + \mathbf{x}_k^\alpha$
      add leaves j and k to T_α while keeping the tree balanced using Algorithm S2
  if $E =$ Transformation **then**
    $j \leftarrow$ a sample from distribution of outcomes of transformation event of i
    $\mathbf{S} \leftarrow \mathbf{S} + \mathbf{x}_j$
    $S \leftarrow S \cup \{j\}$
    for $\alpha \in \mathbf{\Gamma}$ **do**
      $\theta_\alpha \leftarrow \theta_\alpha + \mathbf{x}_j^\alpha$
      add leaf j to T_α while keeping the tree balanced using Algorithm S2
  $\mathbf{S} \leftarrow \mathbf{S} - \mathbf{x}_i$
  $S \leftarrow S - \{i\}$
  for $\alpha \in \mathbf{\Gamma}$ **do**
    $\theta_\alpha \leftarrow \theta_\alpha - \mathbf{x}_i^\alpha$
    remove leaf i from T_α, making the leaf ready for future additions using Algorithm S2

Algorithm S2 Exact algorithm for inserting or removing a leaf from tree T_α keeping it balanced. T_α is represented by a full binary tree where each leaf is labeled with either one entity or $\emptyset$ and each node v has weight w_v equal to the sum of $\mathbf{x}_i^\alpha$ for all leaves under v with label (i) not being $\emptyset$. Assuming a stack S_α keeps all leaves with label $\emptyset$.

```
procedure AddWeight(T_α, i, v, u)
    w_u ← w_u + x_i^α
    if v is under left subtree of u then
        AddWeight(T_α, i, v, the left child of u)
    if v is under right subtree of u then
        AddWeight(T_α, i, v, the right child of u)
procedure InsertLeaf(T_α, i)
    if S_α is empty then
        H ← the height of T_α
        T' ← T_α
        T_α ← a full binary tree of height H + 1, all leaves labeled ∅, and all nodes having weight 0
        replace the left subtree of the root of T_α with T'
        the weight the root of T_α ← the weight of the left child of the root of T_α
        push all leaves under right child of the root of T_α into S_α
    v ← pop one element from S_α
    label of v ← i
    AddWeight(T_α, i, v, the root of T_α)
procedure ReduceWeight(T_α, i, v, u)
    w_u ← w_u + x_i^α
    if v is under left subtree of u then
        ReduceWeight(T_α, i, v, the left child of u)
    if v is under right subtree of u then
        ReduceWeight(T_α, i, v, the right child of u)
procedure RemoveLeaf(T_α, i)
    v ← leaf of T_α with label i
    label of v ← ∅
    push v onto S_α
    ReduceWeight(T_α, i, v, the root of T_α)
```

Recall:

$$\sum_{i,j\in[r]} \left| \kappa \sum_{p\in\mathbf{CDR}} \delta(\zeta_i^{(p)}, \zeta_i^{(p)}) - \delta(\zeta_i^{(p)}, \zeta_j^{(p)}) - \sum_{q=1}^{L_\eta} \left(\delta(\eta_i^{(q)}, \eta_i^{(q)}) - \delta(\eta_i^{(q)}, \eta_j^{(q)}) \right) \right| . \tag{S4}$$

Algorithm S3 Heuristics for choosing target sequences to minimize the objective function (S4).

```
for i ← 2 to r do
    for q ∈ CDR do
        C_i^(q) ← 0
        ζ_i^(q) ← ζ_1^(q)
for p ← 1 to L_η do
    t ← Poisson(κ)
    for u ← 1 to t do
        q ← a uniform random element of CDR where η_1^(p) = ζ_1^(q)
        for i ← 2 to r do
            if η_i^(p) ≠ η_1^(p) then
                C_i^(q) ← C_i^(q) + 1
                ζ_i^(q) ← η_i^(p) with probability 1/C_i^(q)
b ← True
while b = True do
    b ← False
    for i ← 2 to r do
        for q ∈ CDR do
            for s ∈ nucleotide alphabet do
                if replacing ζ_i^(q) with s reduces the objective function then
                    b ← True
                    ζ_i^(q) ← s
```

Algorithm S4 The computeset algoirthm

Let each label be uniformly randomly assigned to an element in a finite Abelian group with large enough order (e.g., 64-bit integers). To compute FNR, FDR, and RF, we just need to compute $|\phi(R)| = |S_R|$, $|\phi(E)| = |S_E|$, and $|\phi(R) \cap \phi(E)| = |S_R \cap S_E|$, where set S_T for tree T can be computed by calling COMPUTESET(T, the root of T).

```
procedure COMPUTESET(T, v)
    w ← the element assigned to the label of v, if v has label; otherwise, w ← 0.
    for u in the children of v do
        w ← w+ COMPUTESET(T, u)
    add element w to set S_T
    return w
```

REFERENCES

Kurosawa Y, Tonegawa S. Organization, structure, and assembly of immunoglobulin heavy chain diversity DNA segments. *The Journal of Experimental Medicine* **155** (1982) 201–218.

doi:10.1084/jem.155.1.201.

Tonegawa S. Somatic generation of antibody diversity. *Nature* **302** (1983) 575–581. doi:10.1038/302575a0.

Neuberger MS, Milstein C. Somatic hypermutation. *Current Opinion in Immunology* **7** (1995) 248–254. doi:10.1016/0952-7915(95)80010-7.

Muramatsu M, Kinoshita K, Fagarasan S, Yamada S, Shinkai Y, Honjo T. Class Switch Recombination and Hypermutation Require Activation-Induced Cytidine Deaminase (AID), a Potential RNA Editing Enzyme. *Cell* **102** (2000) 553–563. doi:10.1016/S0092-8674(00)00078-7.

Mesin L, Schiepers A, Ersching J, Barbulescu A, Cavazzoni CB, Angelini A, et al. Restricted clonality and limited germinal center reentry characterize memory b cell reactivation by boosting. *Cell* **180** (2020) 92–106.e11. doi:https://doi.org/10.1016/j.cell.2019.11.032.

Hsiao YC, Shang Y, DiCara DM, Yee A, Lai J, Kim SH, et al. Immune repertoire mining for rapid affinity optimization of mouse monoclonal antibodies. *mAbs* **11** (2019) 735–746. doi:10.1080/19420862.2019.1584517.

Safonova Y, Pevzner P. IgEvolution: clonal analysis of antibody repertoires. *BioRxiv* (2019) 1–18. doi:10.1101/725424.

Peled JU, Kuang FL, Iglesias-Ussel MD, Roa S, Kalis SL, Goodman MF, et al. The biochemistry of somatic hypermutation. *Annu. Rev. Immunol.* **26** (2008) 481–511.

Rogozin I, Kolchanov N. Somatic hypermutagenesis in immunoglobulin genes. ii. influence of neighbouring base sequences on mutagenesis. *Biochim Biophys Acta* **1171** (1992) 11–18.

Rogozin IB, Diaz M. Cutting edge: DGYW/WRCH is a better predictor of mutability at G:C bases in Ig hypermutation than the widely accepted RGYW/WRCY motif and probably reflects a two-step activation-induced cytidine deaminase-triggered process. *J. Immunol.* **172** (2004) 3382–3384.

Bransteitter R, Pham P, Calabrese P, Goodman MF. Biochemical analysis of hypermutational targeting by wild type and mutant activation-induced cytidine deaminase. *J. Biol. Chem.* **279** (2004) 51612–51621.

Smith DS, Creadon G, Jena PK, Portanova JP, Kotzin BL, Wysocki LJ. Di- and trinucleotide target preferences of somatic mutagenesis in normal and autoreactive B cells. *J. Immunol.* **156** (1996) 2642–2652.

Shapiro GS, Ellison MC, Wysocki LJ. Sequence-specific targeting of two bases on both DNA strands by the somatic hypermutation mechanism. *Mol. Immunol.* **40** (2003) 287–295.

Yaari G, Vander Heiden JA, Uduman M, Gadala-Maria D, Gupta N, Joel JN, et al. Models of somatic hypermutation targeting and substitution based on synonymous mutations from high-throughput immunoglobulin sequencing data. *Frontiers in Immunology* **4** (2013). doi:10.3389/fimmu.2013.00358.

Robinson D, Foulds L. Comparison of phylogenetic trees. *Mathematical Biosciences* **53** (1981) 131–147.
